# Supplementary material for: The Determination of Assistance-as-Needed Support by an Ankle–Foot Orthosis for Patients with Foot Drop
Source: Int J Environ Res Public Health. 2023 Aug 30;20(17):6687. doi: 10.3390/ijerph20176687 (PMC10487717; doi:10.3390/ijerph20176687)
Supplement: Supplementary file 1 [file ijerph-20-06687-s001.zip › ijerph-2441422-supplementary.pdf]

Supplementary Material

Determination of assistance-as-needed support by an ankle-foot orthosis for patients with foot drop

David Scherb*, Patrick Steck, Iris Wechsler, Sandro Wartzack and Jörg Miehling

Engineering Design, Friedrich-Alexander-Universität Erlangen-Nürnberg, 91058 Erlangen, Germany
* Correspondence: scherb@mfk.fau.de

Figure S1

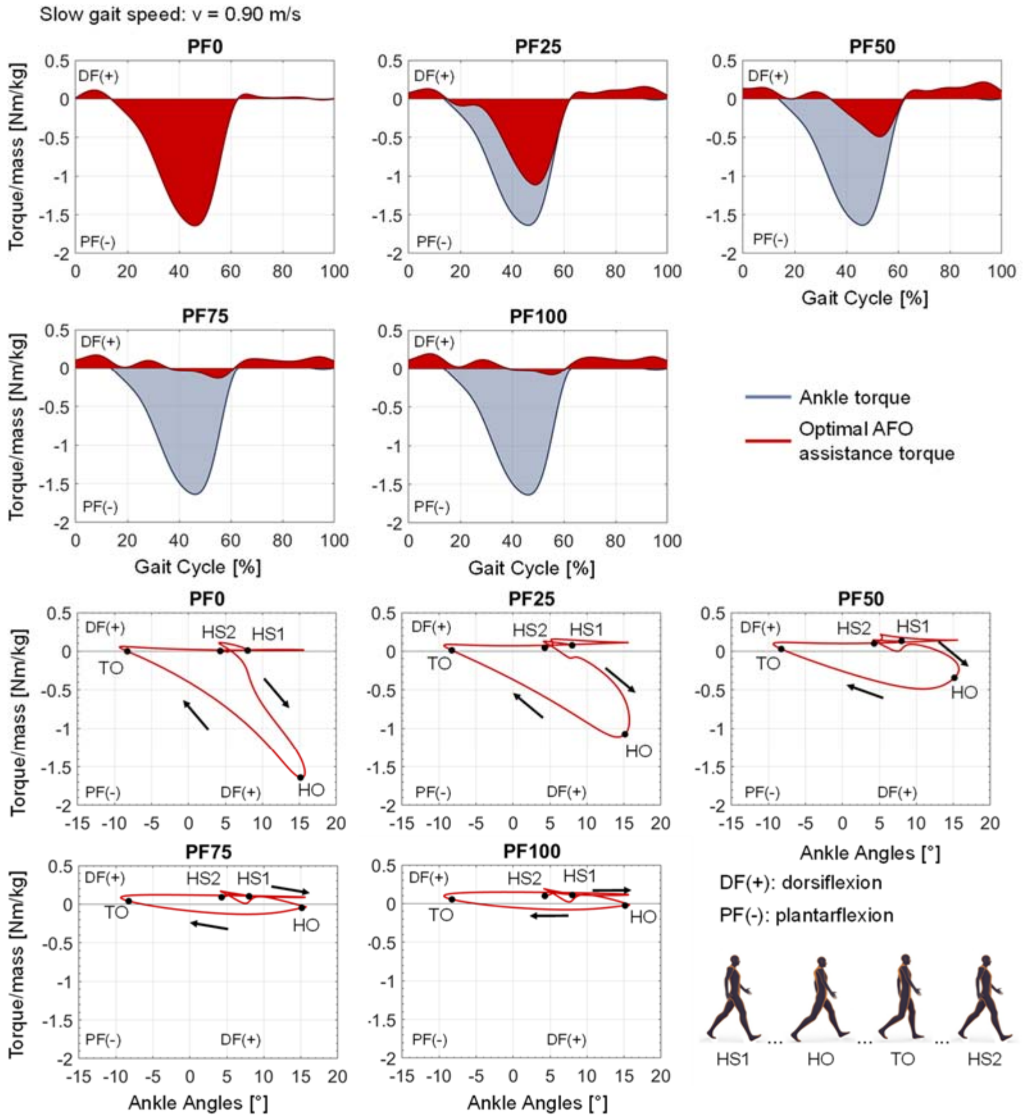

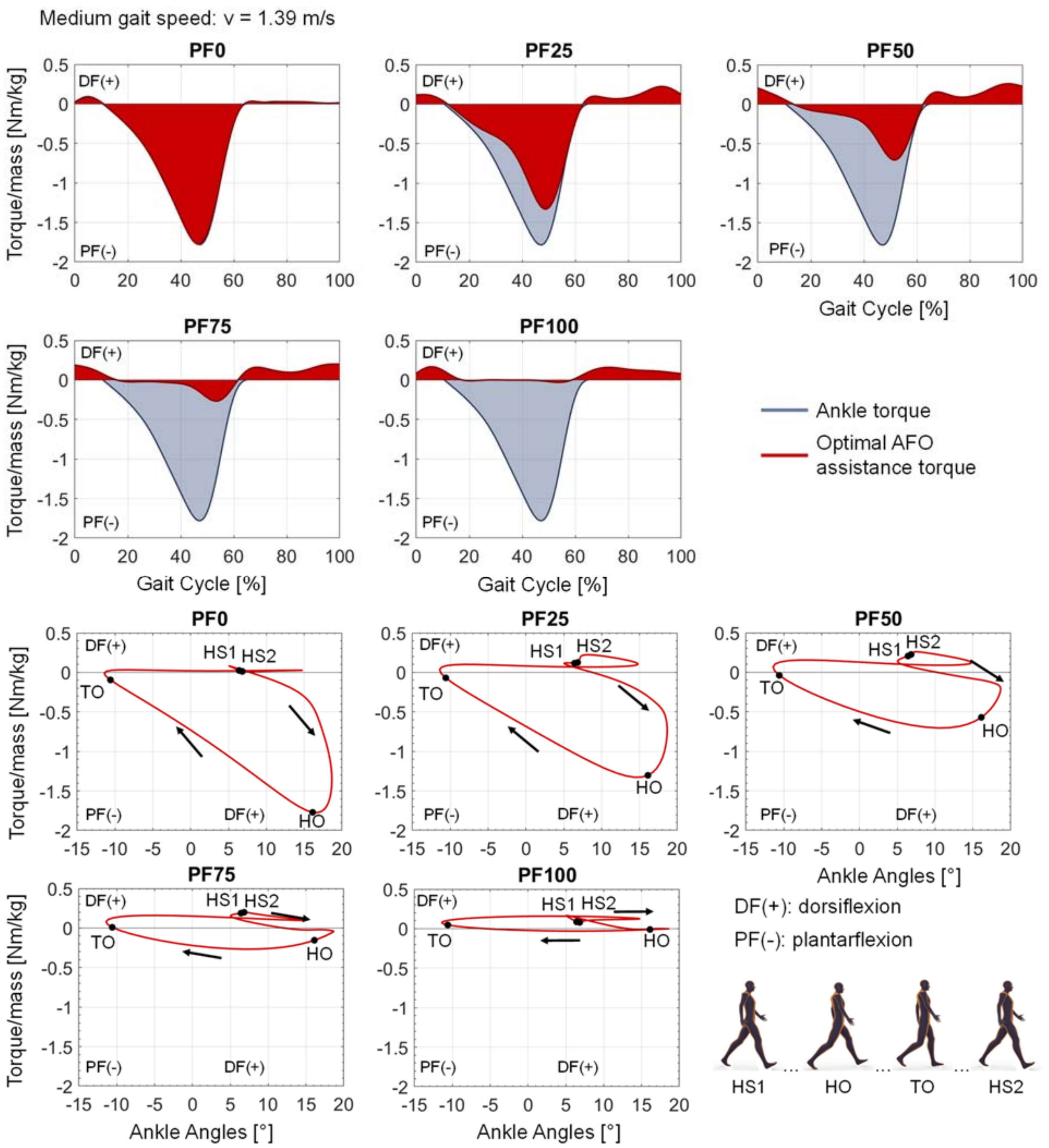

Medium gait speed: v = 1.39 m/s
PF0
PF25
PF50
PF75
PF100
Torque/mass [Nm/kg]
DF(+)
PF(-)
Gait Cycle [%]
Ankle torque
Optimal AFO assistance torque
HS1
HS2
TO
HO
Ankle Angles [°]
DF(+): dorsiflexion
PF(-): plantarflexion

Figure S2

Figure S3

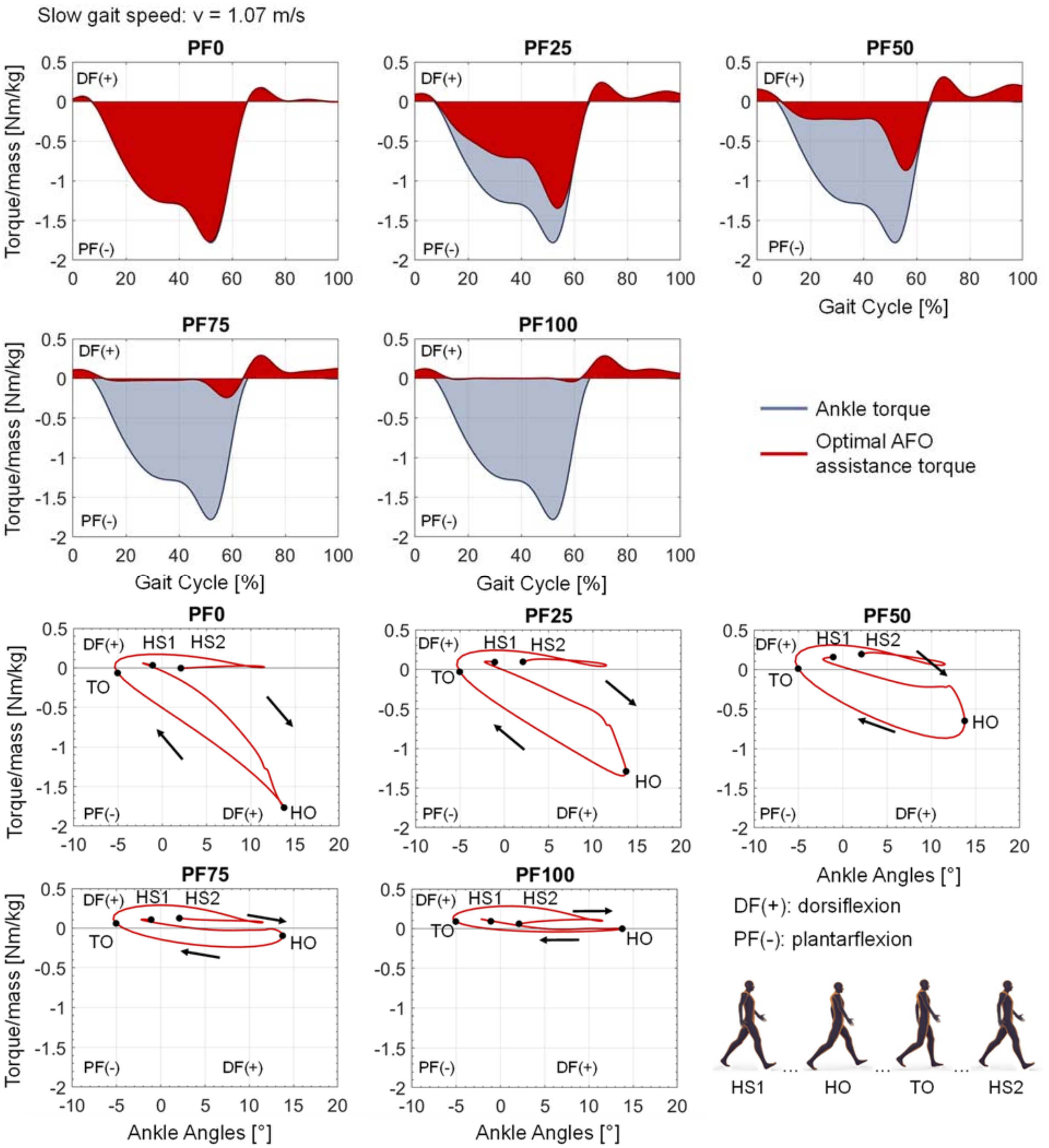

Slow gait speed: v = 1.07 m/s
PF0
PF25
PF50
PF75
PF100
Torque/mass [Nm/kg]
Gait Cycle [%]
DF(+)
PF(-)
Ankle torque
Optimal AFO assistance torque
HS1
HS2
TO
HO
Ankle Angles [°]
DF(+): dorsiflexion
PF(-): plantarflexion

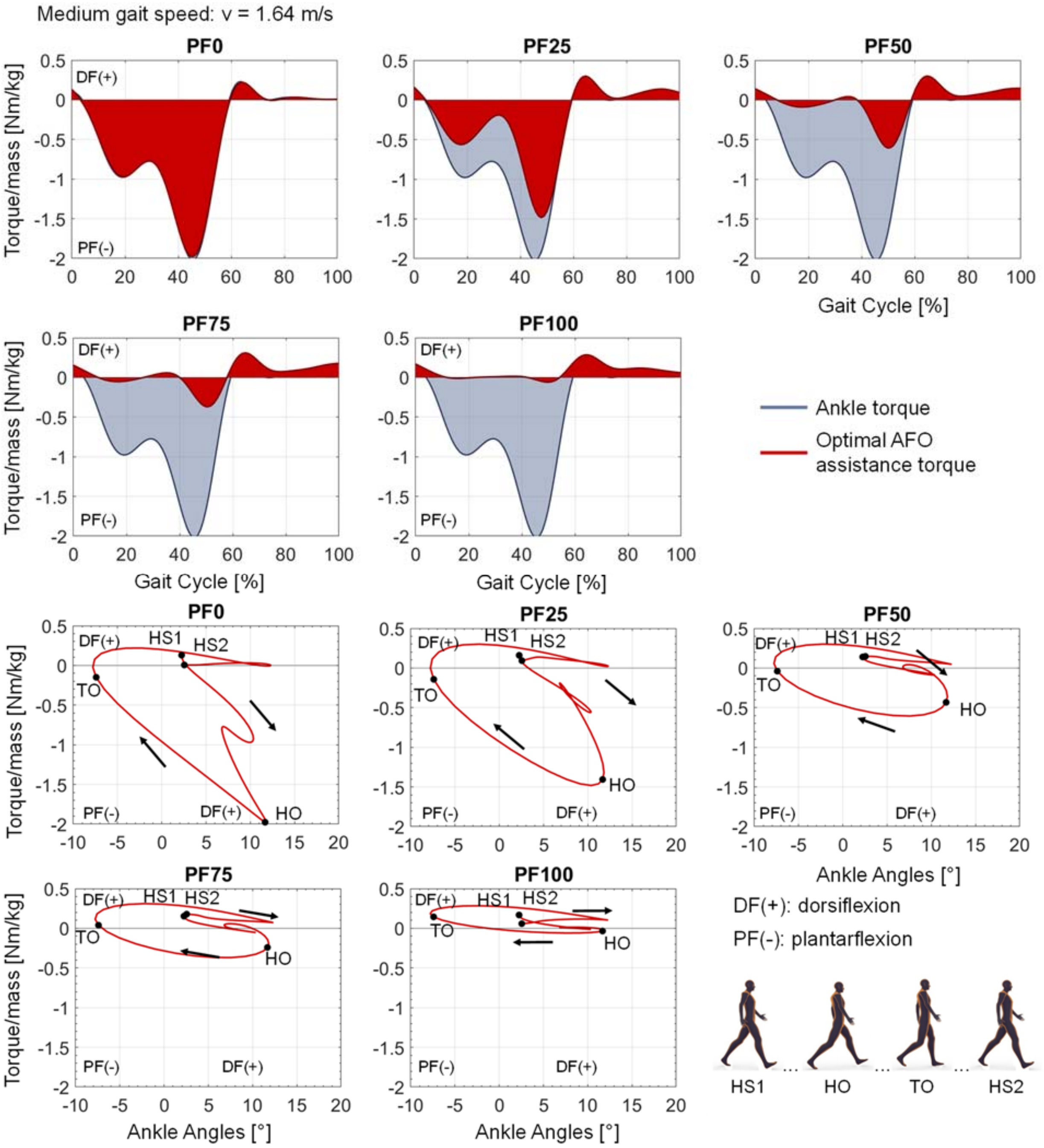

Medium gait speed: v = 1.64 m/s
PF0
PF25
PF50
PF75
PF100
Torque/mass [Nm/kg]
Gait Cycle [%]
DF(+)
PF(-)
Ankle torque
Optimal AFO assistance torque
HS1
HS2
TO
HO
Ankle Angles [°]
DF(+): dorsiflexion
PF(-): plantarflexion

Figure S4

Figure S5

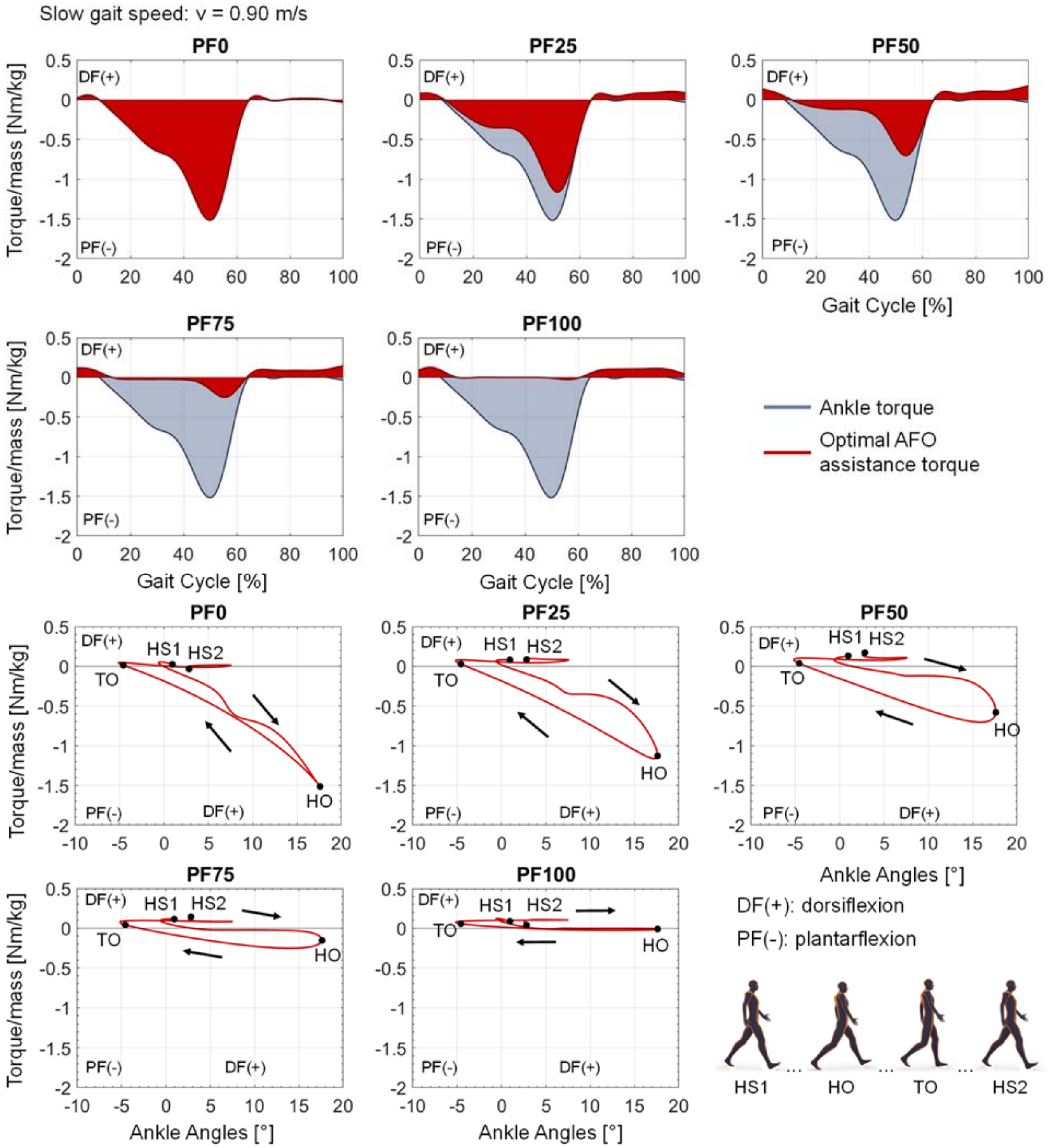

Slow gait speed: v = 0.90 m/s
PF0
PF25
PF50
PF75
PF100
Torque/mass [Nm/kg]
Gait Cycle [%]
DF(+)
PF(-)
Ankle torque
Optimal AFO assistance torque
HS1
HS2
TO
HO
Ankle Angles [°]
DF(+): dorsiflexion
PF(-): plantarflexion

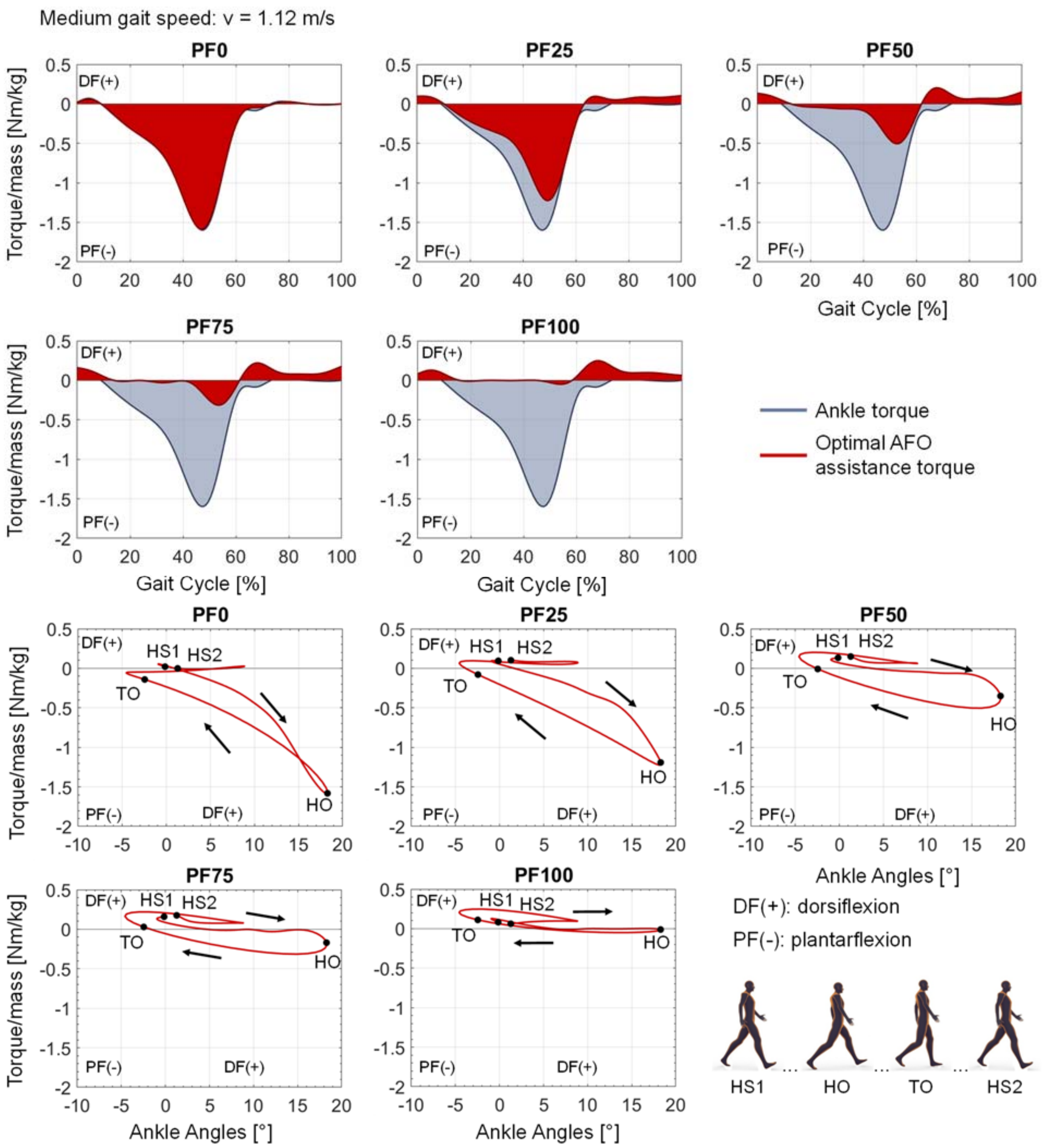
Medium gait speed: v = 1.12 m/s
PF0
PF25
PF50
PF75
PF100
Torque/mass [Nm/kg]
DF(+)
PF(-)
Gait Cycle [%]
Ankle torque
Optimal AFO assistance torque
HS1
HS2
TO
HO
Ankle Angles [°]
DF(+): dorsiflexion
PF(-): plantarflexion

Figure S6

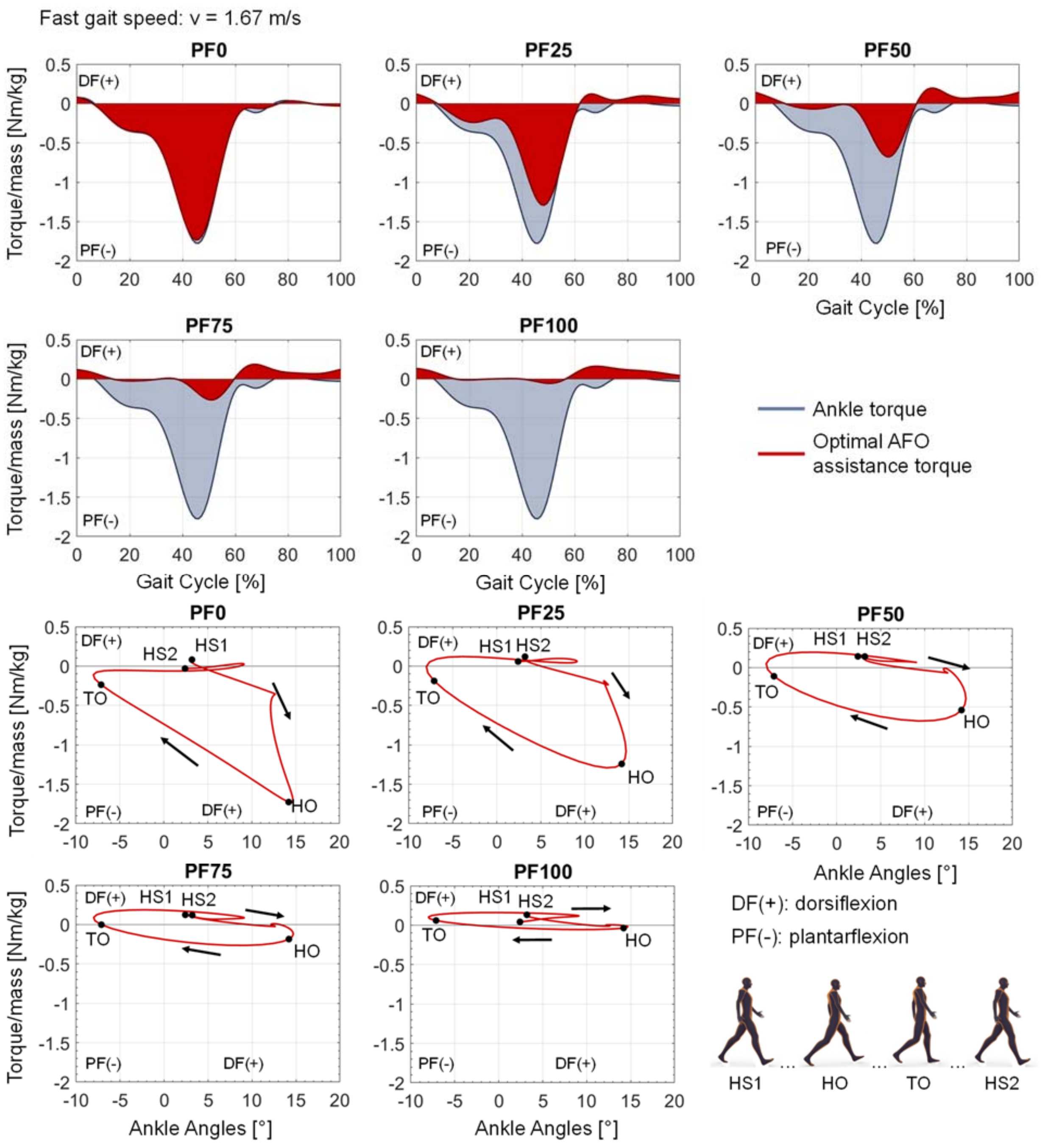
Fast gait speed: v = 1.67 m/s
PF0
PF25
PF50
PF75
PF100
Torque/mass [Nm/kg]
Gait Cycle [%]
DF(+)
PF(-)
Ankle torque
Optimal AFO assistance torque
HS1
HS2
TO
HO
Ankle Angles [°]
DF(+): dorsiflexion
PF(-): plantarflexion
HS1
HO
TO
HS2

Figure S7

Figure S8

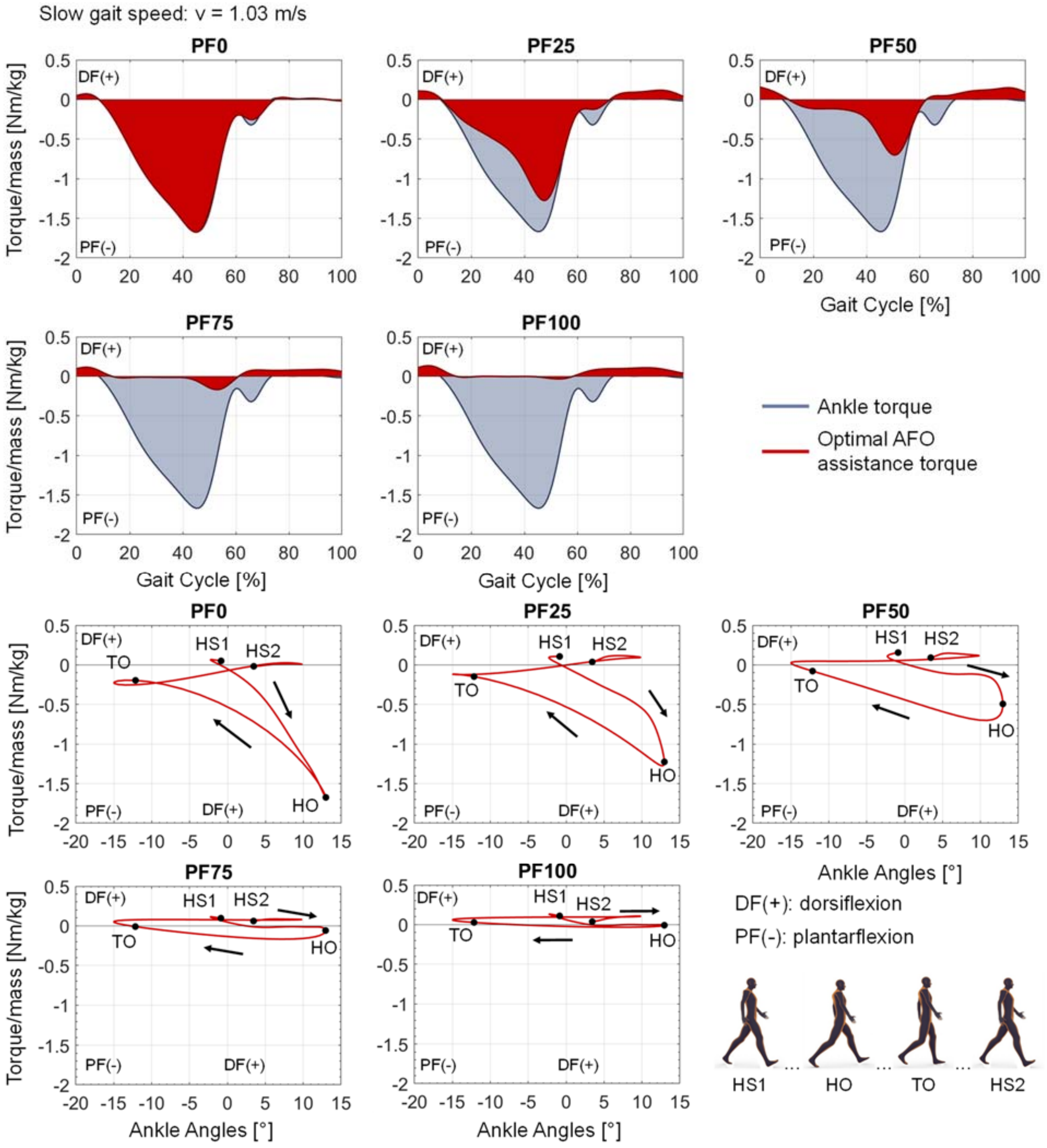

Slow gait speed: v = 1.03 m/s
PF0
PF25
PF50
PF75
PF100
Torque/mass [Nm/kg]
DF(+)
PF(-)
Gait Cycle [%]
Ankle torque
Optimal AFO assistance torque
HS1
HS2
TO
HO
Ankle Angles [°]
DF(+): dorsiflexion
PF(-): plantarflexion
HS1
HO
TO
HS2

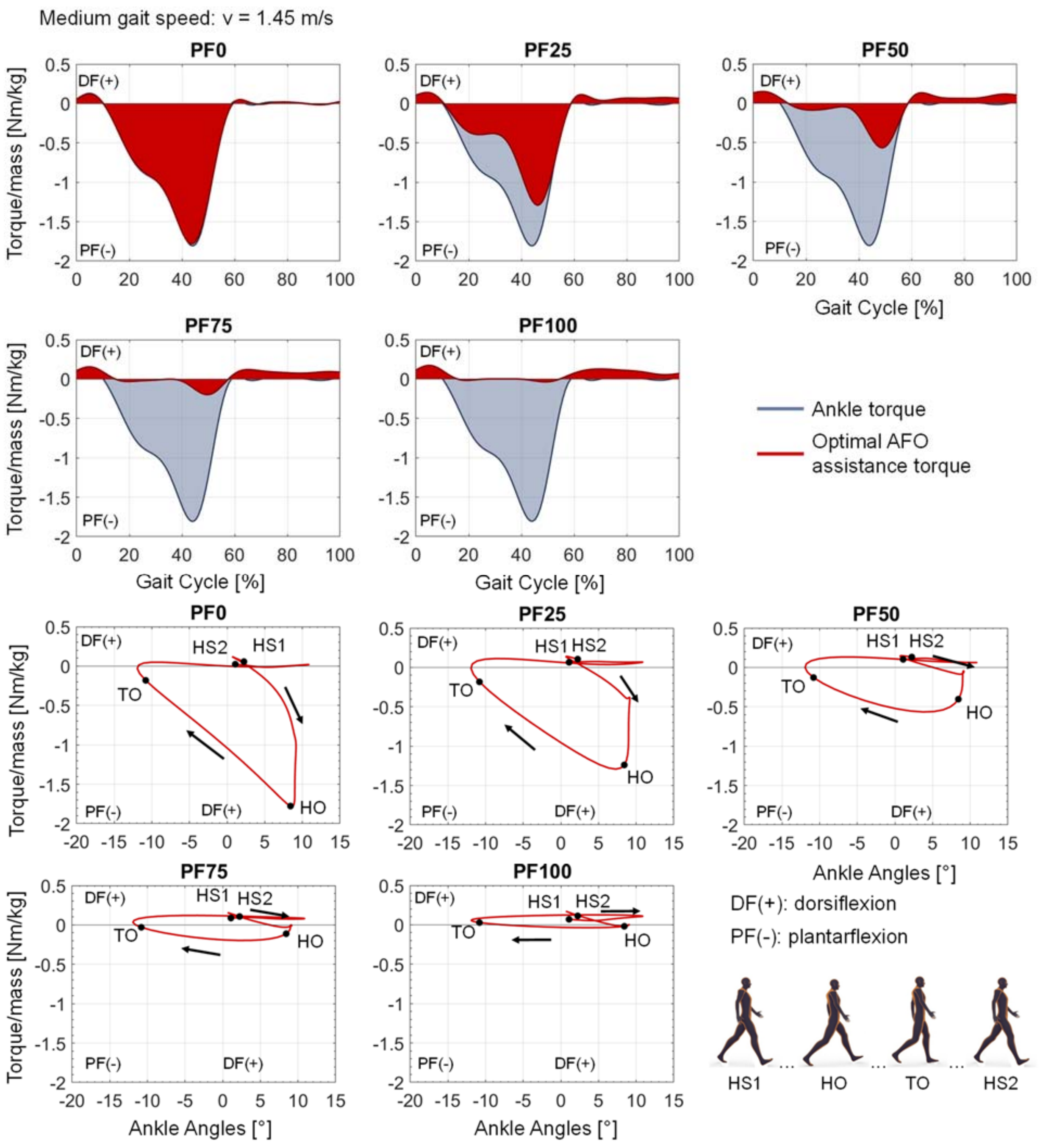
Medium gait speed: v = 1.45 m/s
PF0
PF25
PF50
PF75
PF100
DF(+)
PF(-)
Torque/mass [Nm/kg]
Gait Cycle [%]
Ankle torque
Optimal AFO assistance torque
HS1
HS2
TO
HO
Ankle Angles [°]
DF(+): dorsiflexion
PF(-): plantarflexion

Figure S9

Figure S10

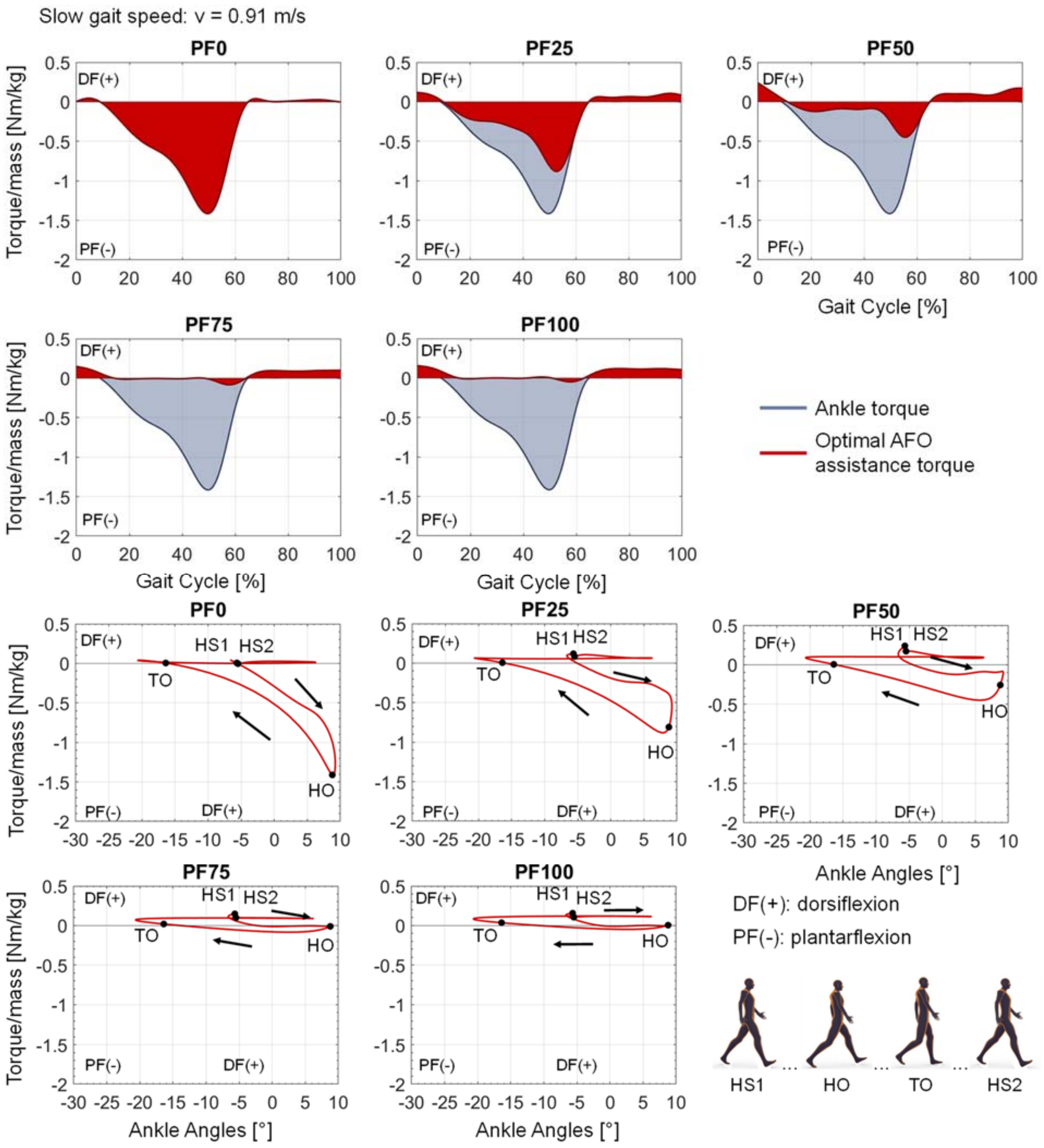

Slow gait speed: v = 0.91 m/s
PF0
PF25
PF50
PF75
PF100
Torque/mass [Nm/kg]
Gait Cycle [%]
DF(+)
PF(-)
Ankle torque
Optimal AFO assistance torque
HS1
HS2
TO
HO
Ankle Angles [°]
DF(+): dorsiflexion
PF(-): plantarflexion

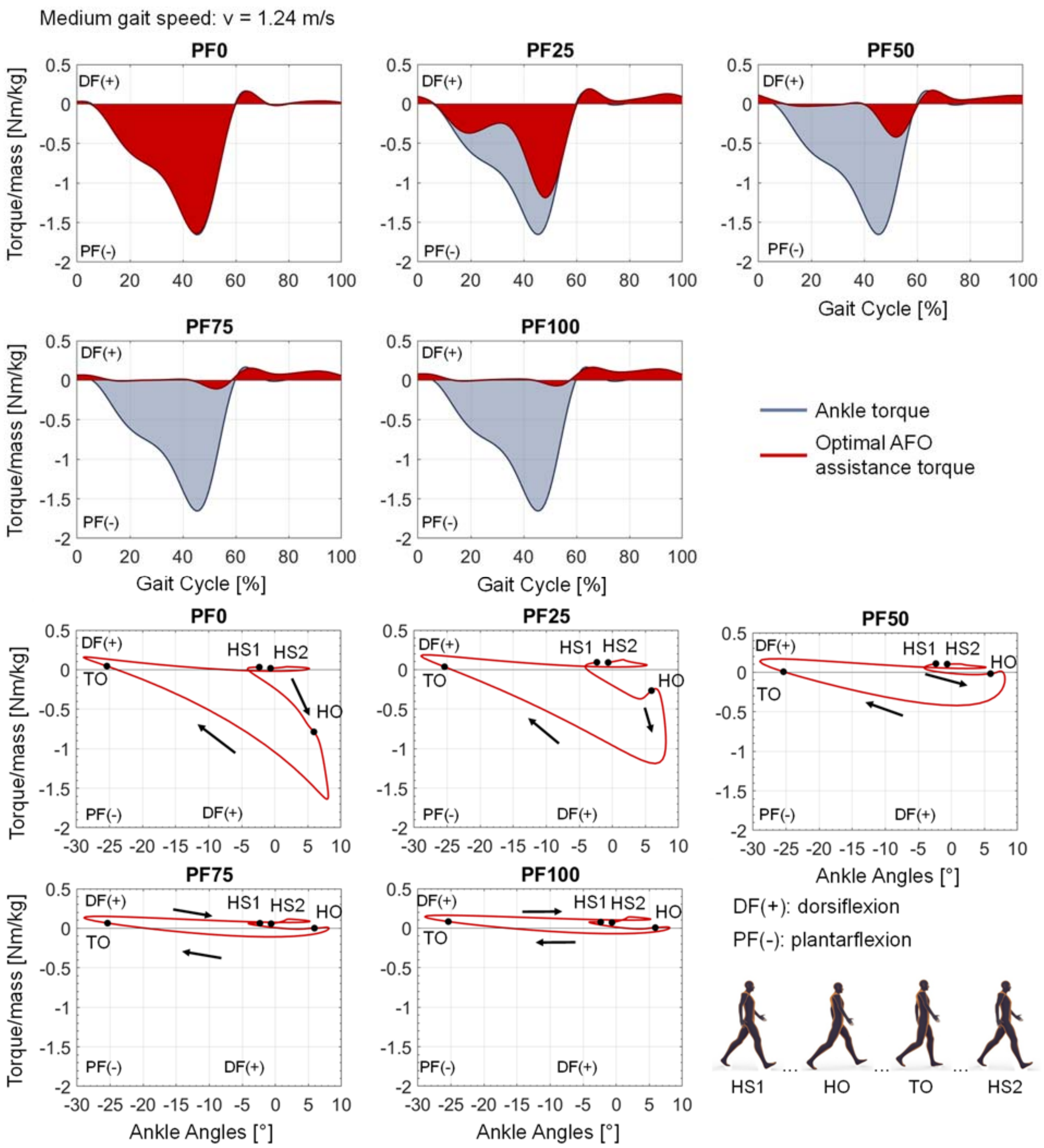

Medium gait speed: v = 1.24 m/s
PF0
PF25
PF50
PF75
PF100
Torque/mass [Nm/kg]
DF(+)
PF(-)
Gait Cycle [%]
Ankle torque
Optimal AFO assistance torque
HS1
HS2
HO
TO
Ankle Angles [°]
DF(+): dorsiflexion
PF(-): plantarflexion

Figure S11

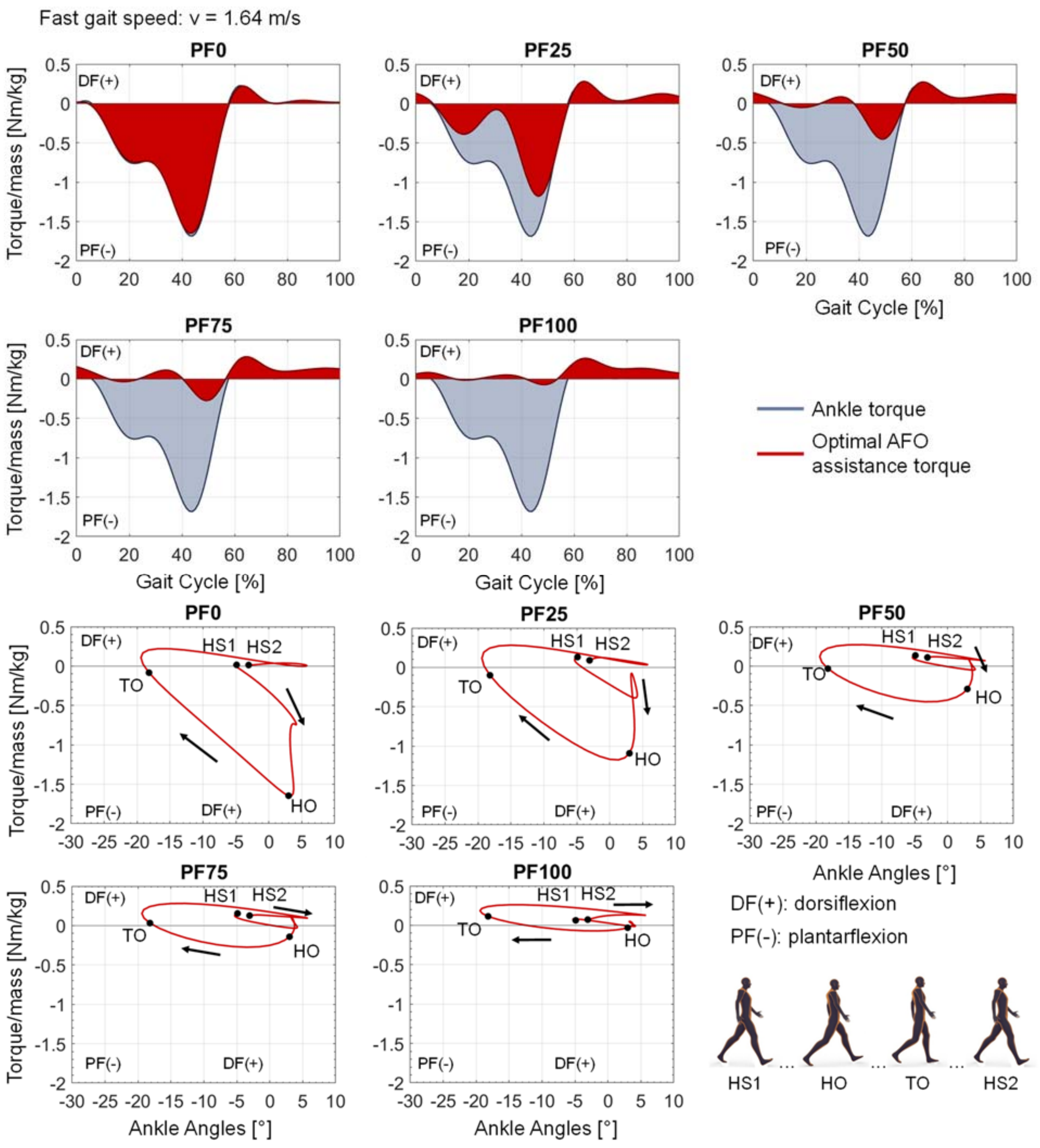

Fast gait speed: v = 1.64 m/s
PF0
PF25
PF50
PF75
PF100
DF(+)
PF(-)
Torque/mass [Nm/kg]
Gait Cycle [%]
Ankle torque
Optimal AFO assistance torque
HS1
HS2
TO
HO
Ankle Angles [°]
DF(+): dorsiflexion
PF(-): plantarflexion

Figure S12

Figure S13

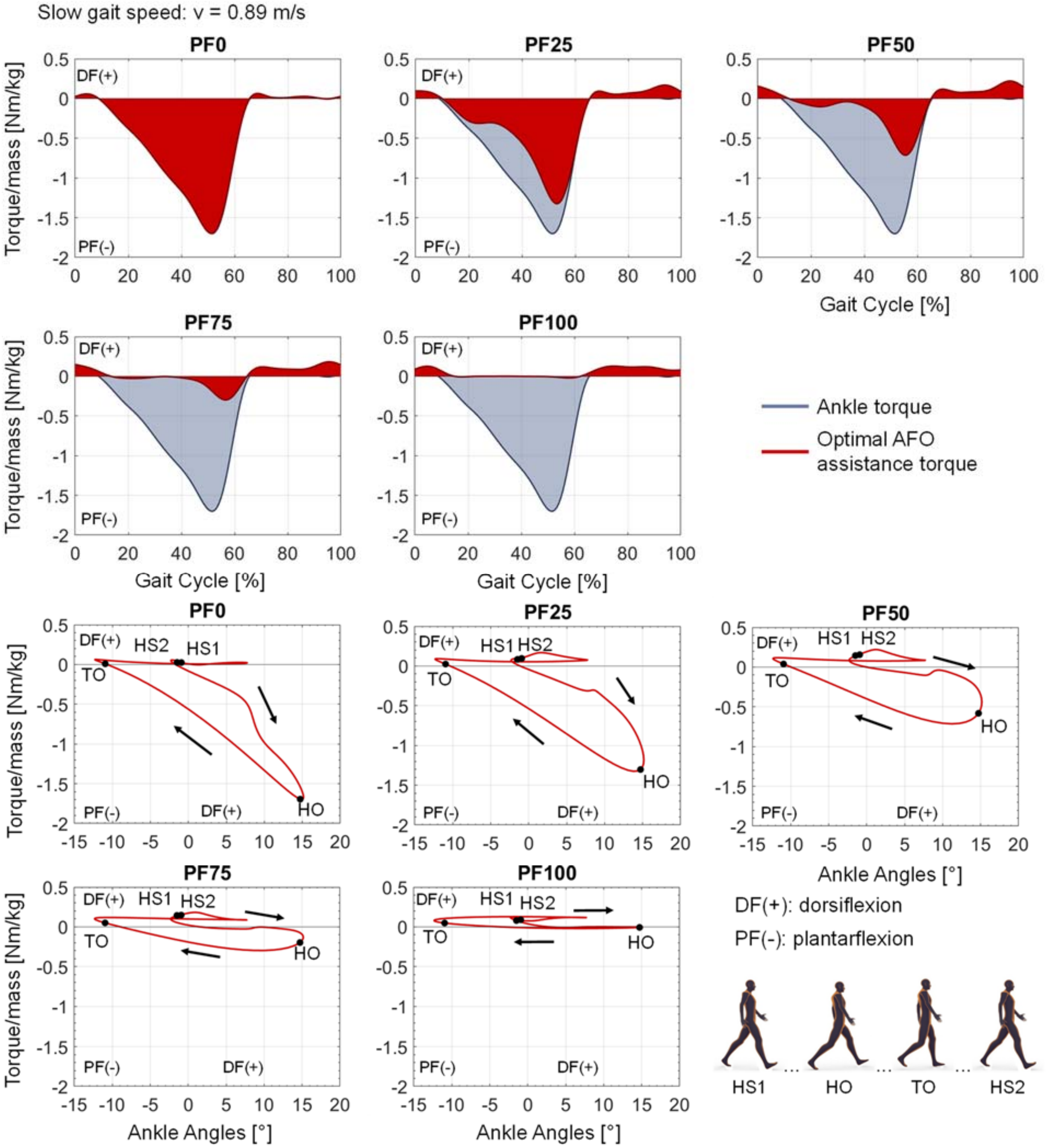

Slow gait speed: v = 0.89 m/s
PF0
PF25
PF50
PF75
PF100
Torque/mass [Nm/kg]
Gait Cycle [%]
DF(+)
PF(-)
Ankle torque
Optimal AFO assistance torque
HS1
HS2
TO
HO
Ankle Angles [°]
DF(+): dorsiflexion
PF(-): plantarflexion
HS1 ... HO ... TO ... HS2

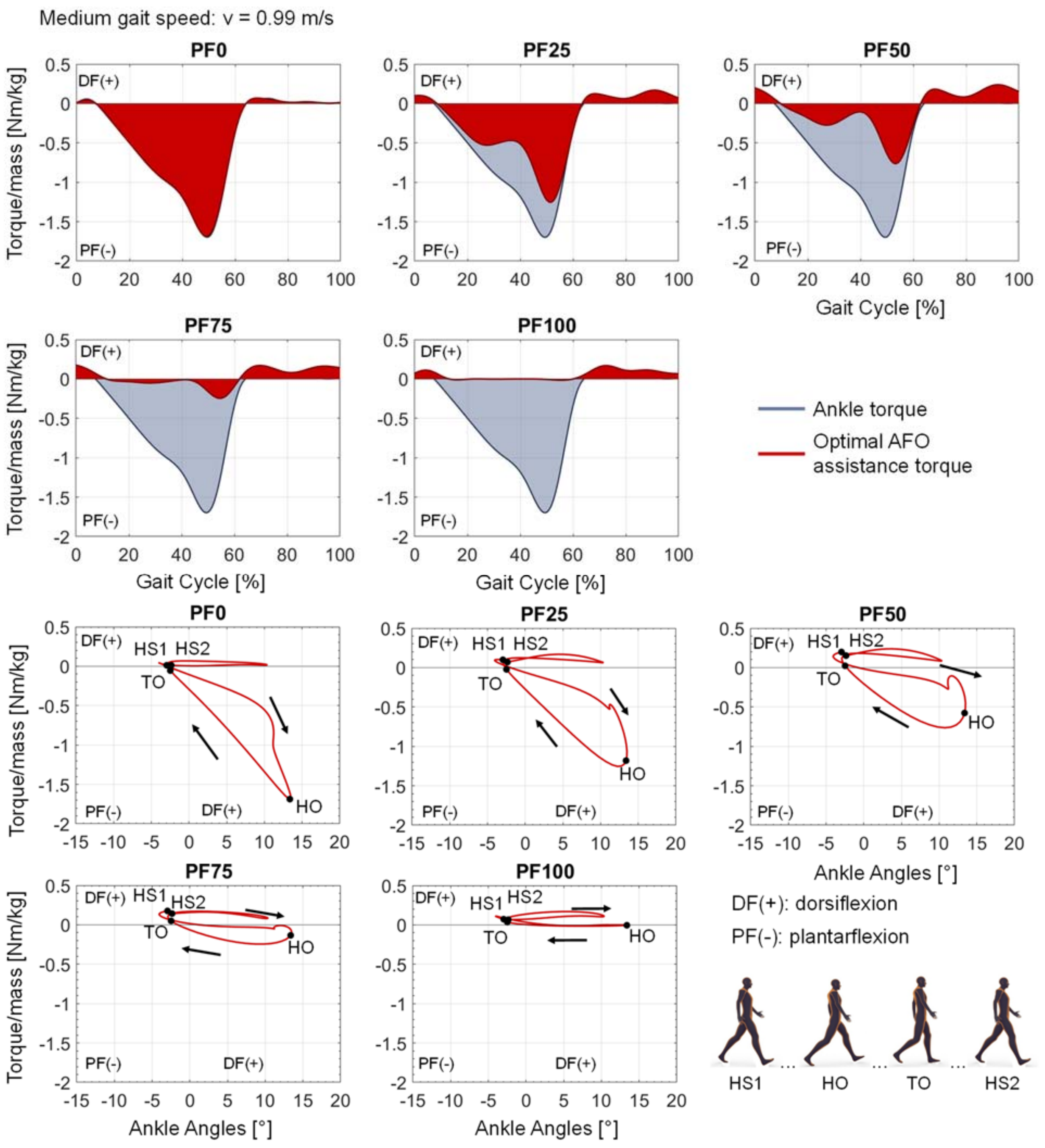

Medium gait speed: v = 0.99 m/s
PF0
PF25
PF50
PF75
PF100
Torque/mass [Nm/kg]
DF(+)
PF(-)
Gait Cycle [%]
Ankle torque
Optimal AFO assistance torque
HS1
HS2
TO
HO
Ankle Angles [°]
DF(+): dorsiflexion
PF(-): plantarflexion

Figure S14

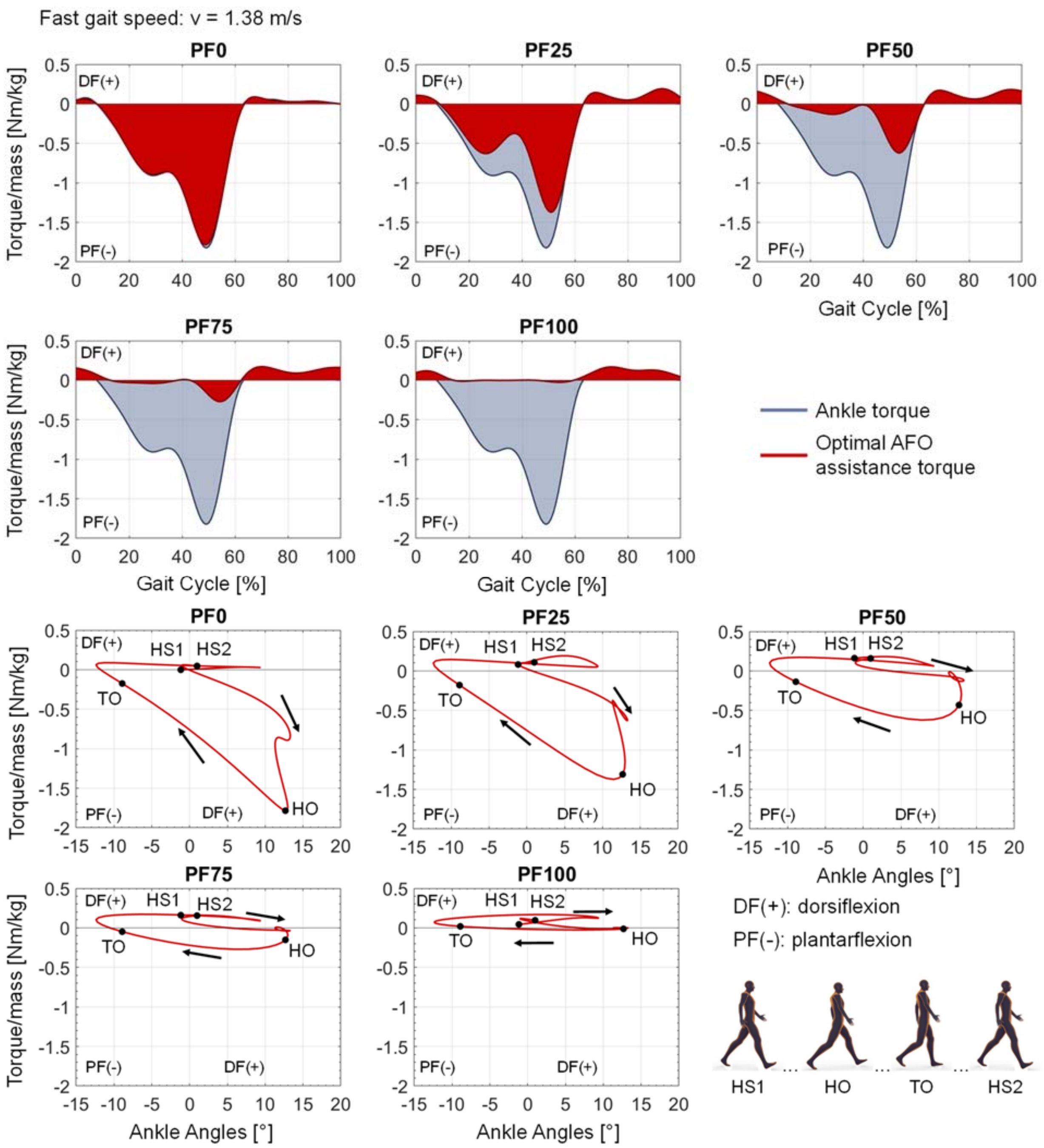
Fast gait speed: v = 1.38 m/s
PF0
PF25
PF50
PF75
PF100
Torque/mass [Nm/kg]
Gait Cycle [%]
DF(+)
PF(-)
Ankle torque
Optimal AFO assistance torque
HS1
HS2
TO
HO
Ankle Angles [°]
DF(+): dorsiflexion
PF(-): plantarflexion

Figure S15

Figure S16

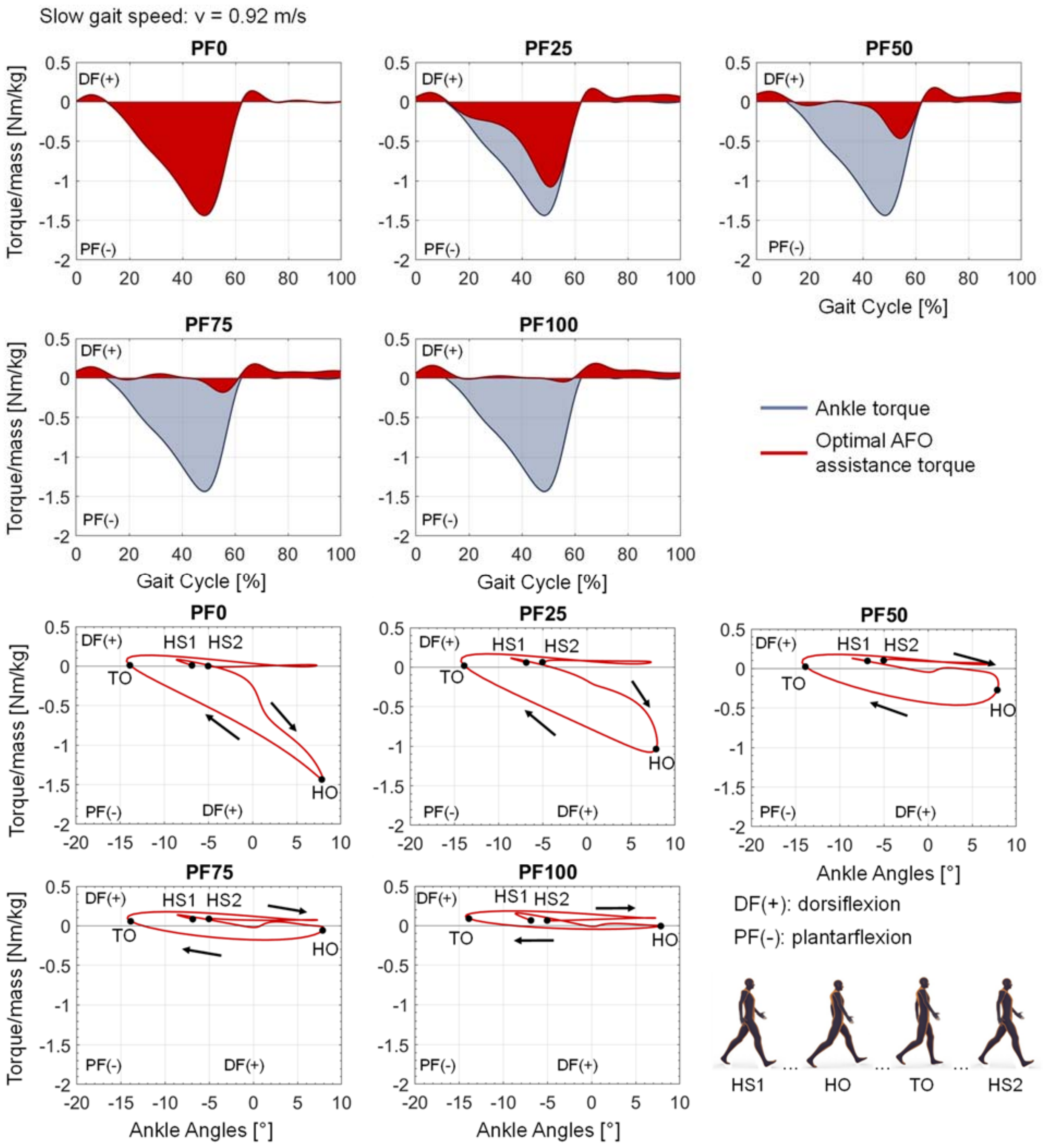

Slow gait speed: v = 0.92 m/s
PF0
PF25
PF50
PF75
PF100
Torque/mass [Nm/kg]
Gait Cycle [%]
DF(+)
PF(-)
Ankle torque
Optimal AFO assistance torque
HS1
HS2
TO
HO
Ankle Angles [°]
DF(+): dorsiflexion
PF(-): plantarflexion
HS1 ... HO ... TO ... HS2

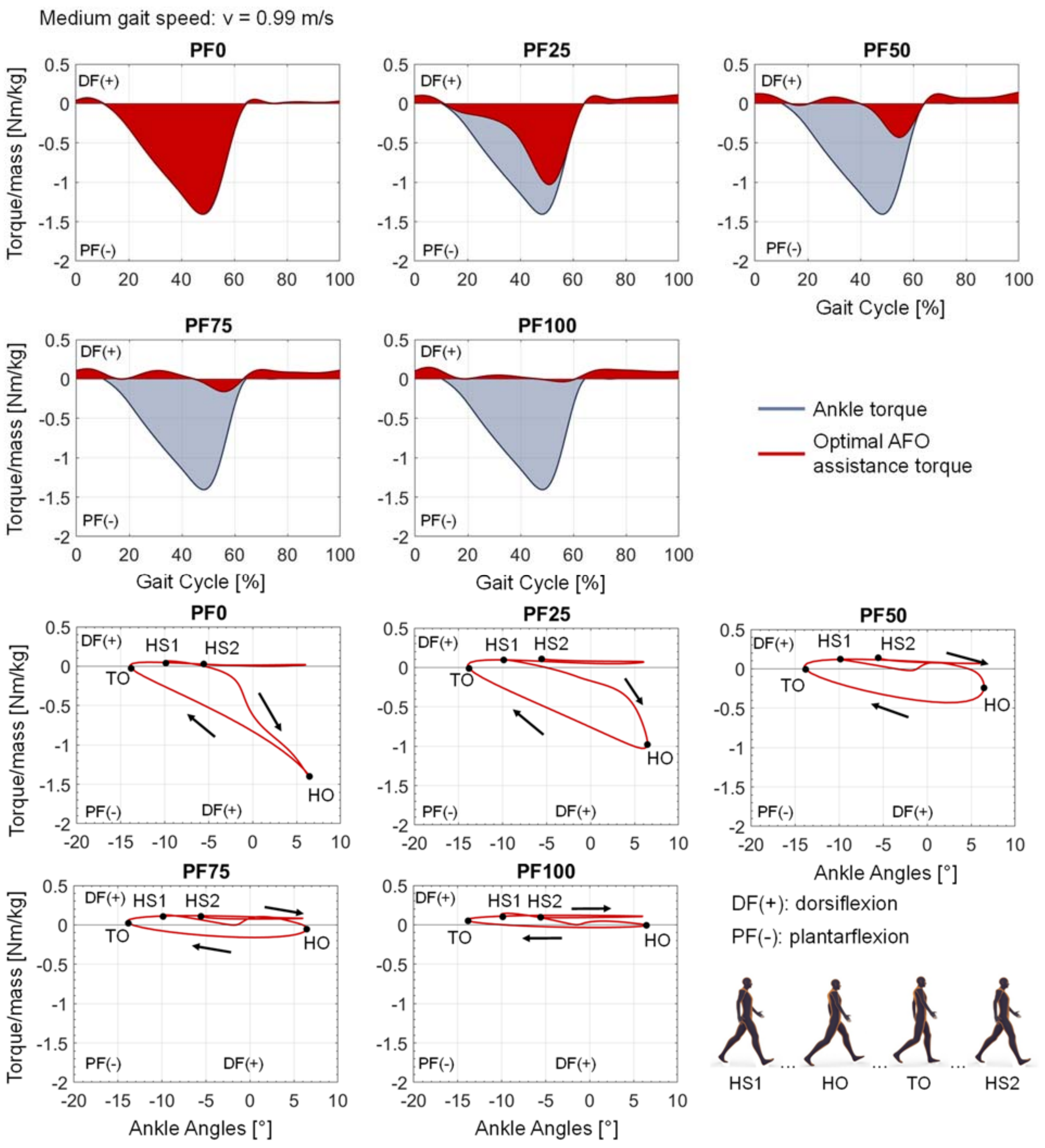

Medium gait speed: v = 0.99 m/s
PF0
PF25
PF50
PF75
PF100
DF(+)
PF(-)
Torque/mass [Nm/kg]
Gait Cycle [%]
Ankle torque
Optimal AFO assistance torque
HS1
HS2
TO
HO
Ankle Angles [°]
DF(+): dorsiflexion
PF(-): plantarflexion

Figure S17

Figure S18

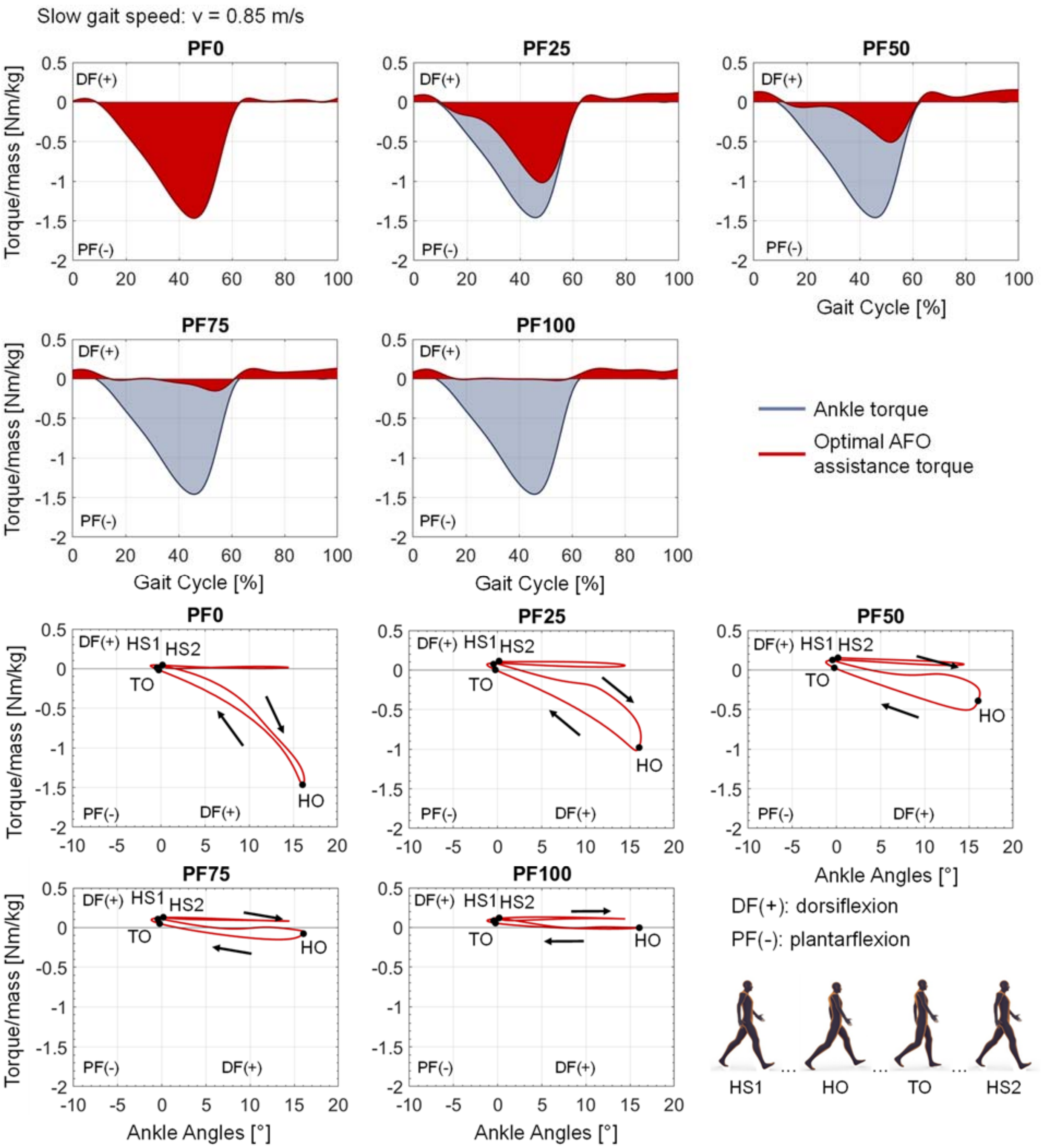

Slow gait speed: v = 0.85 m/s
PF0
PF25
PF50
PF75
PF100
Torque/mass [Nm/kg]
Gait Cycle [%]
DF(+)
PF(-)
Ankle torque
Optimal AFO assistance torque
HS1
HS2
TO
HO
Ankle Angles [°]
DF(+): dorsiflexion
PF(-): plantarflexion
HS1
HO
TO
HS2

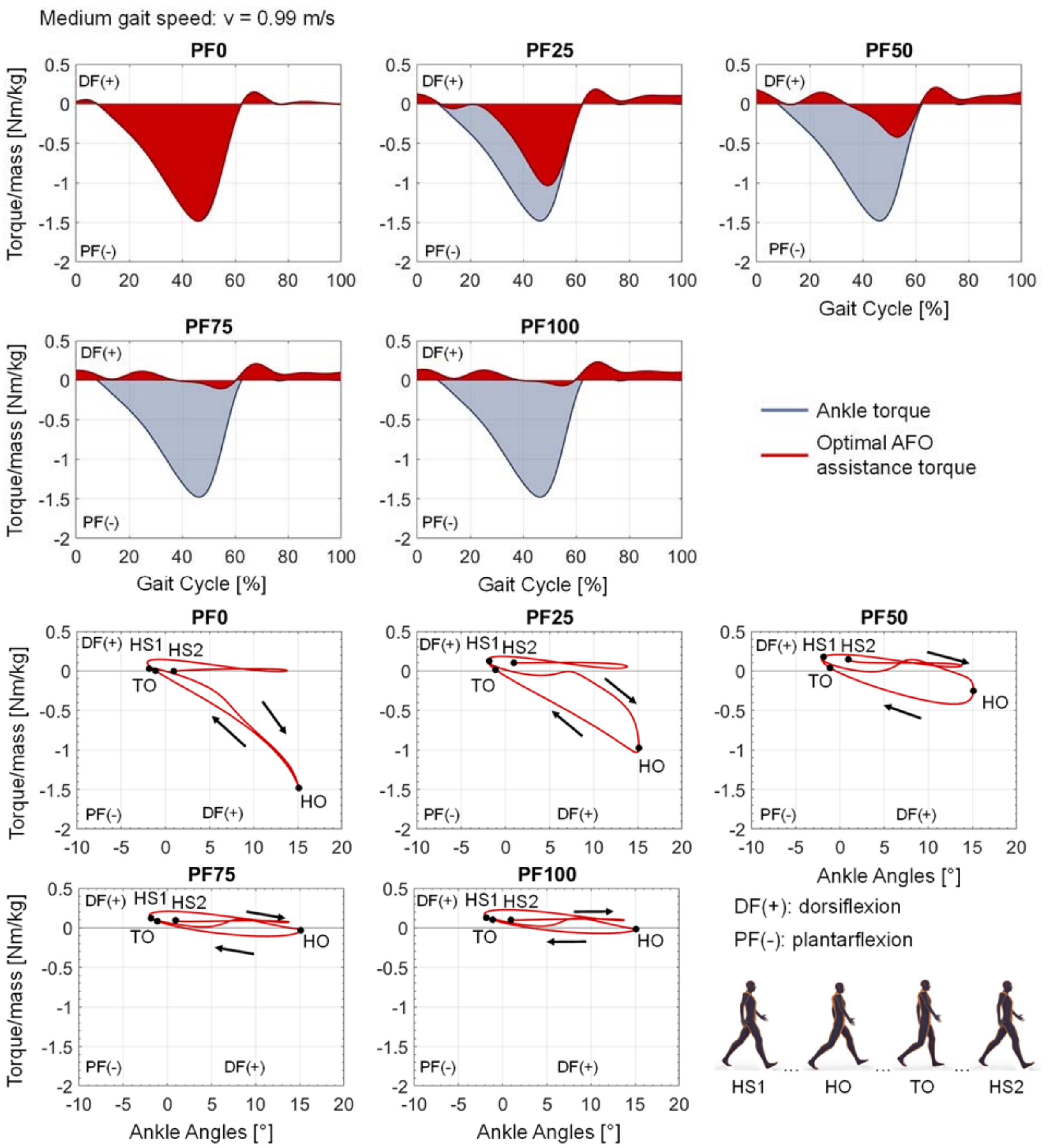

Medium gait speed: v = 0.99 m/s
PF0
PF25
PF50
PF75
PF100
Torque/mass [Nm/kg]
DF(+)
PF(-)
Gait Cycle [%]
Ankle torque
Optimal AFO assistance torque
HS1
HS2
TO
HO
Ankle Angles [°]
DF(+): dorsiflexion
PF(-): plantarflexion
HS1 ... HO ... TO ... HS2

Figure S19

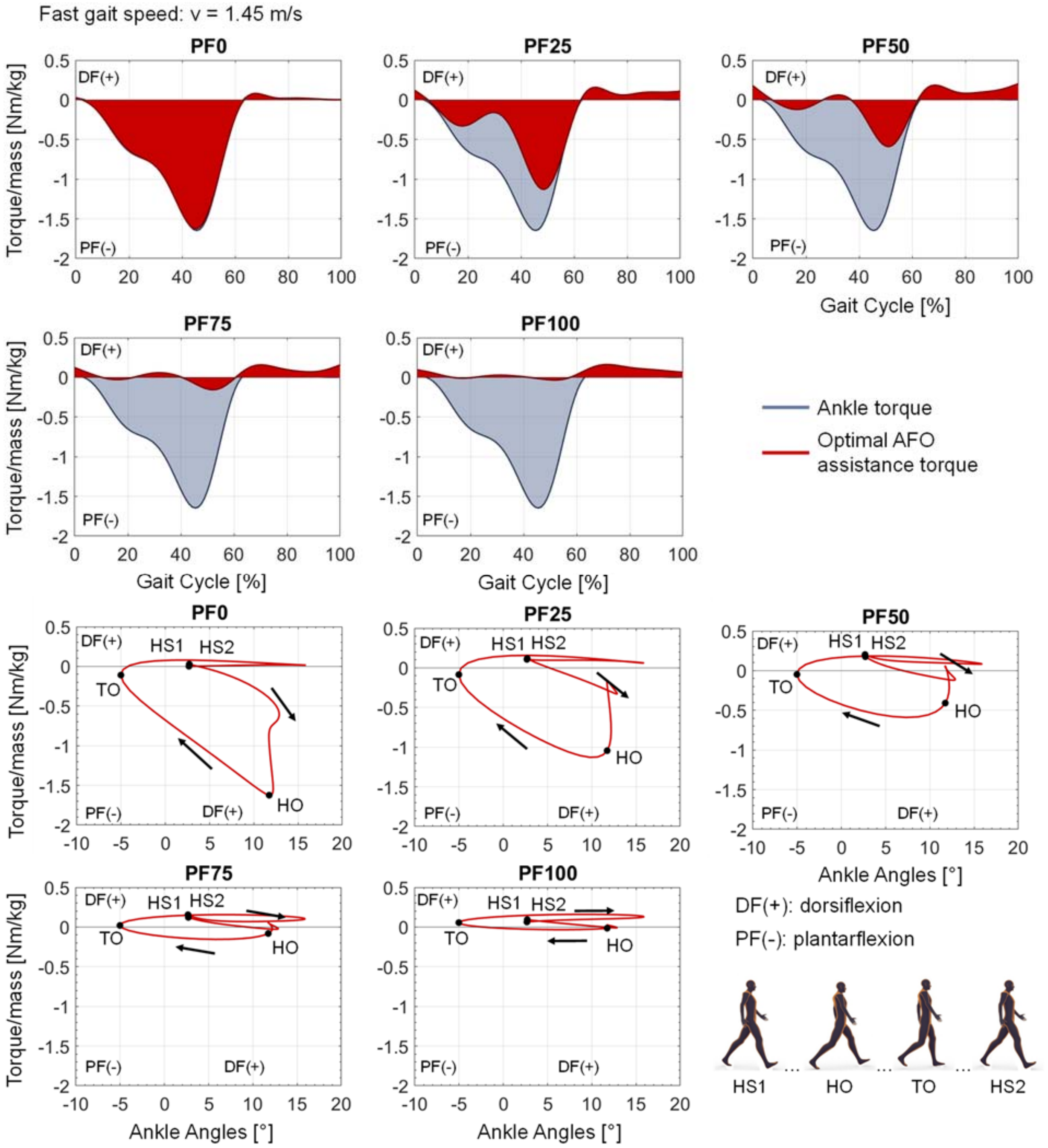

Fast gait speed: v = 1.45 m/s
PF0
PF25
PF50
PF75
PF100
DF(+)
PF(-)
Torque/mass [Nm/kg]
Gait Cycle [%]
Ankle torque
Optimal AFO assistance torque
HS1
HS2
TO
HO
Ankle Angles [°]
DF(+): dorsiflexion
PF(-): plantarflexion

Figure S20

Figure S21

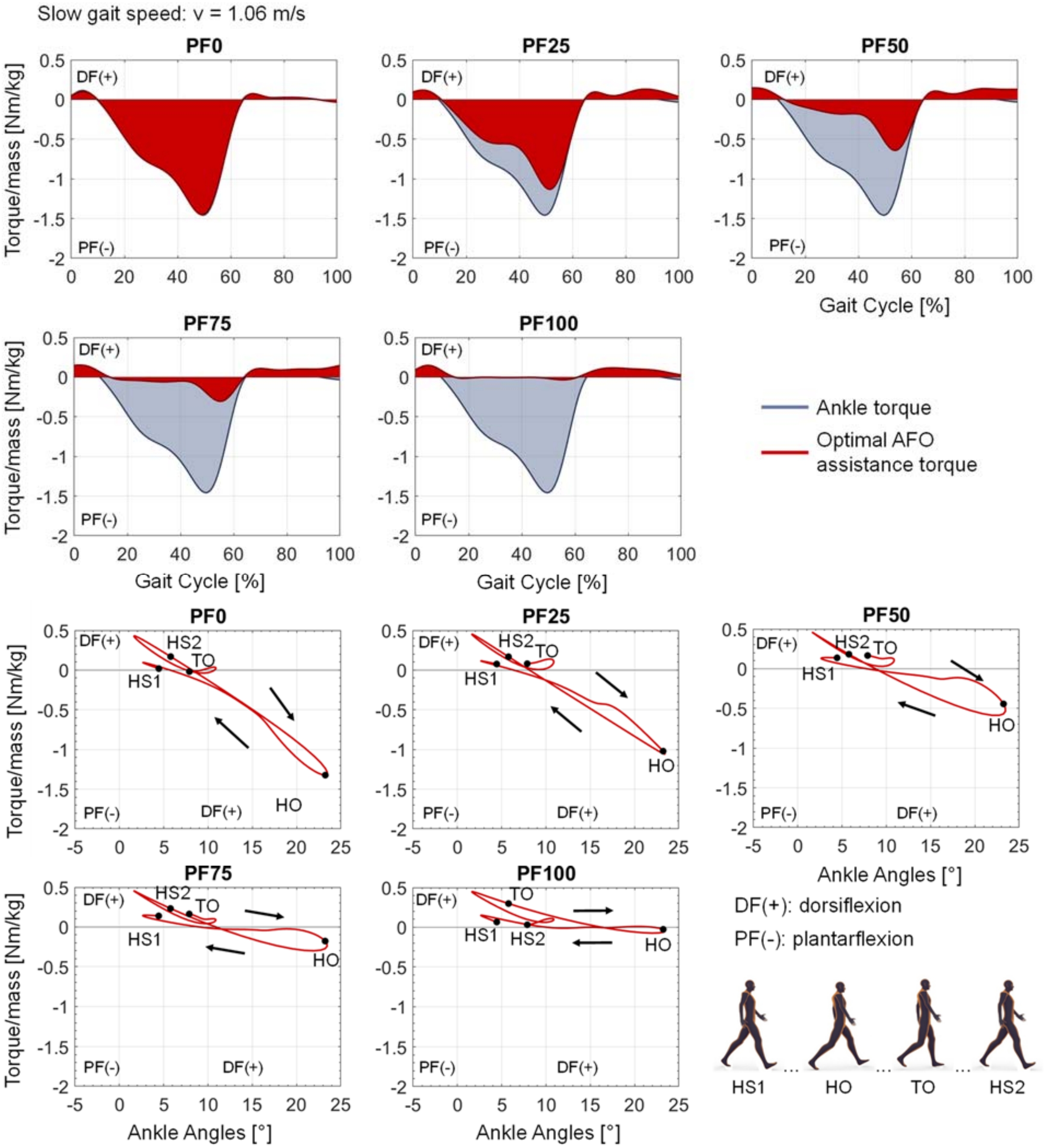

Slow gait speed: v = 1.06 m/s
PF0
PF25
PF50
PF75
PF100
Torque/mass [Nm/kg]
Gait Cycle [%]
DF(+)
PF(-)
Ankle torque
Optimal AFO assistance torque
Ankle Angles [°]
HS1
HS2
TO
HO
DF(+): dorsiflexion
PF(-): plantarflexion

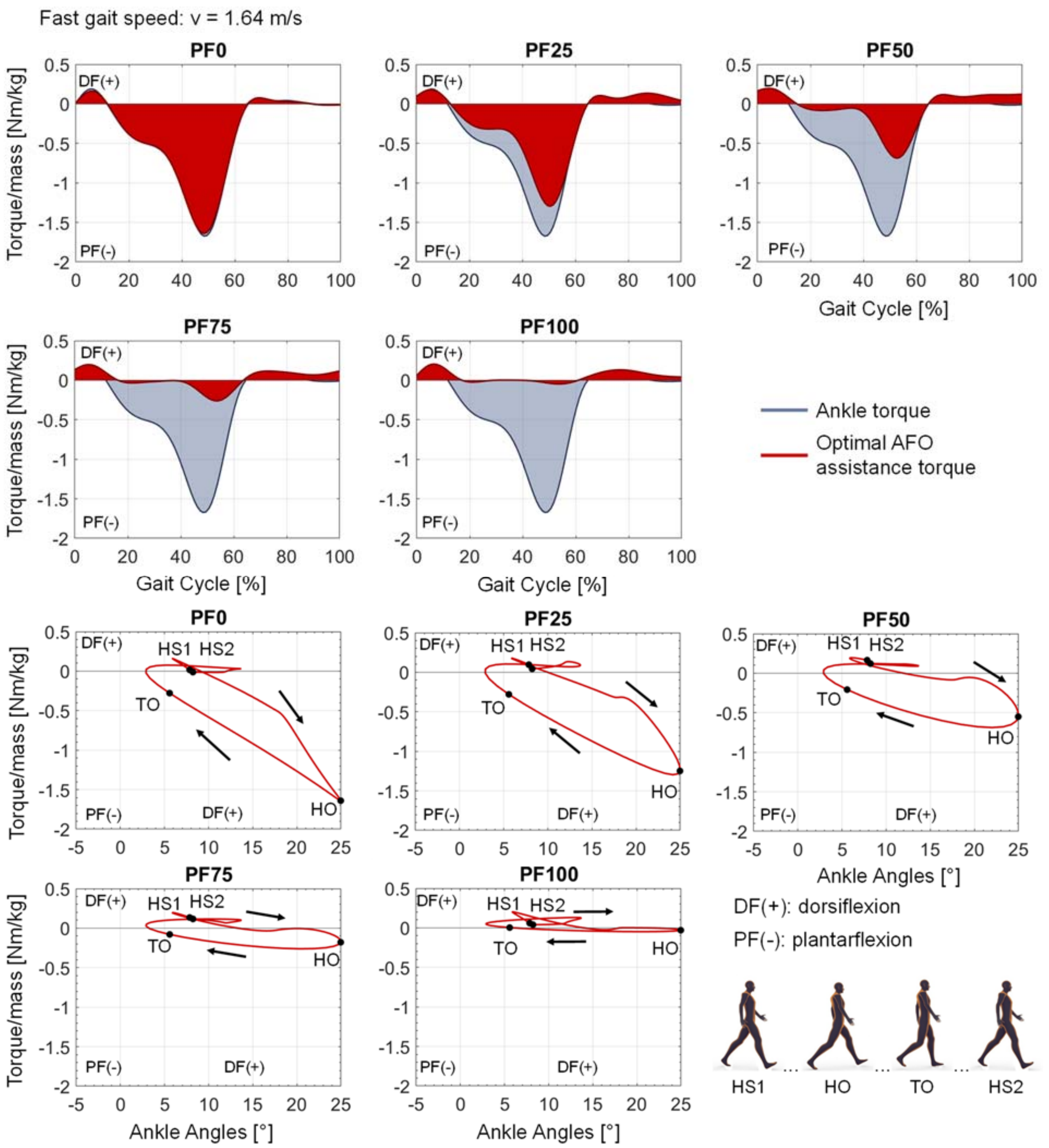

Fast gait speed: v = 1.64 m/s
PF0
PF25
PF50
PF75
PF100
Torque/mass [Nm/kg]
DF(+)
PF(-)
Gait Cycle [%]
Ankle torque
Optimal AFO assistance torque
HS1
HS2
TO
HO
Ankle Angles [°]
DF(+): dorsiflexion
PF(-): plantarflexion

Figure S22

Figure S23

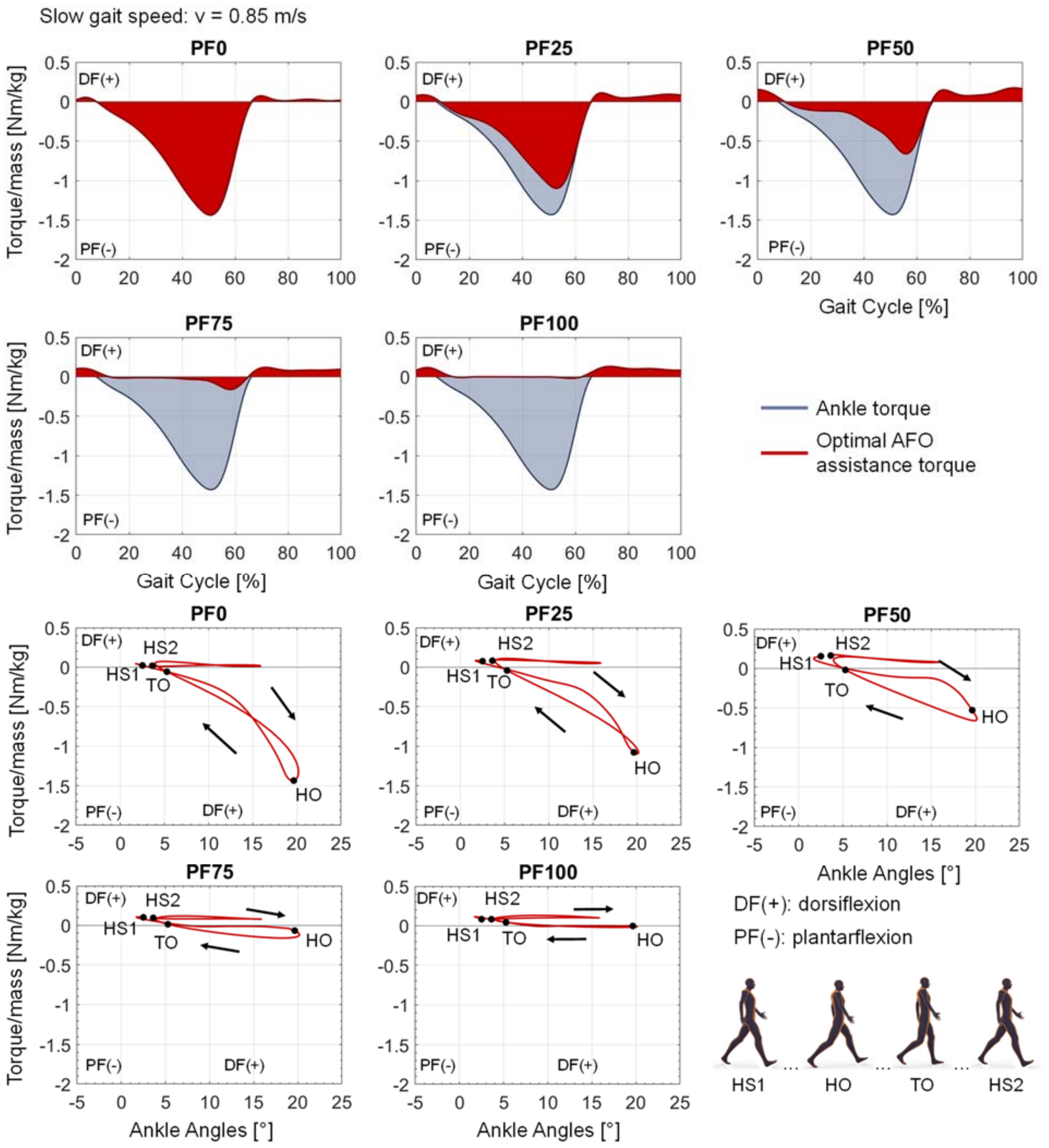

Slow gait speed: v = 0.85 m/s
PF0
PF25
PF50
PF75
PF100
DF(+)
PF(-)
Torque/mass [Nm/kg]
Gait Cycle [%]
Ankle torque
Optimal AFO assistance torque
HS1
HS2
TO
HO
Ankle Angles [°]
DF(+): dorsiflexion
PF(-): plantarflexion
HS1 ... HO ... TO ... HS2

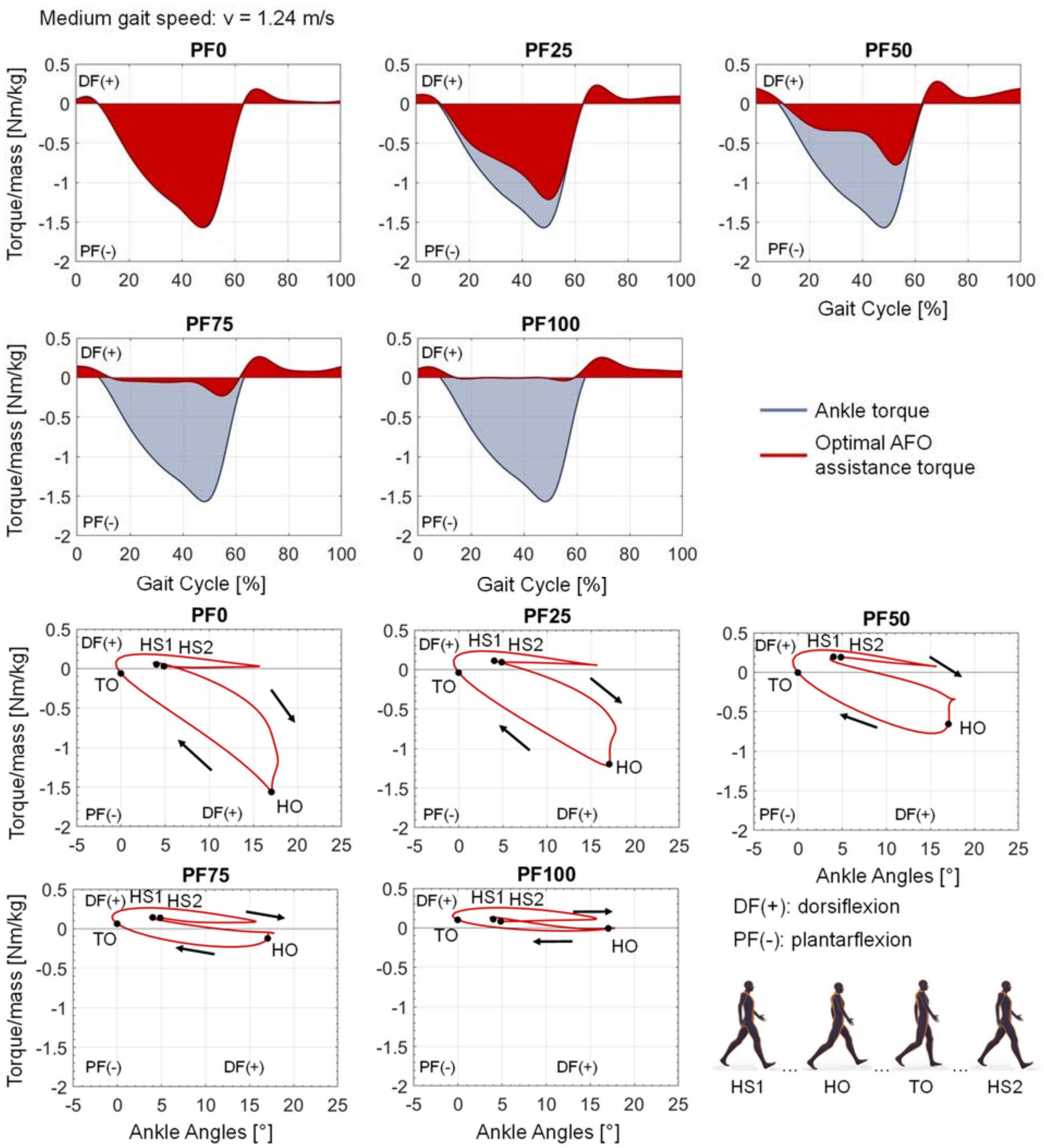

Medium gait speed: v = 1.24 m/s
PF0
PF25
PF50
PF75
PF100
Torque/mass [Nm/kg]
Gait Cycle [%]
DF(+)
PF(-)
Ankle torque
Optimal AFO assistance torque
HS1
HS2
TO
HO
Ankle Angles [°]
DF(+): dorsiflexion
PF(-): plantarflexion

Figure S24

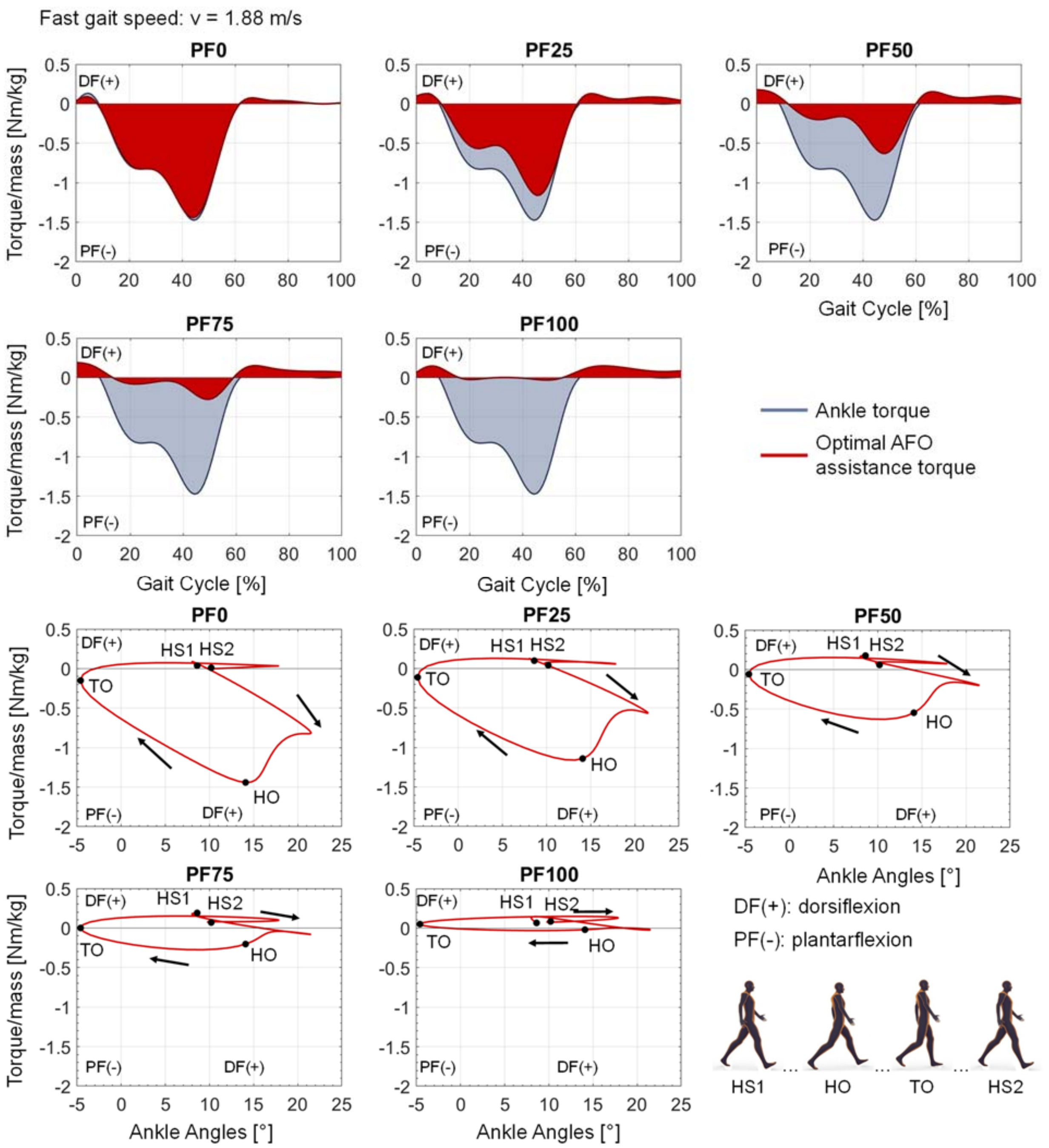
Fast gait speed: v = 1.88 m/s
PF0
PF25
PF50
PF75
PF100
Torque/mass [Nm/kg]
Gait Cycle [%]
DF(+)
PF(-)
Ankle torque
Optimal AFO assistance torque
HS1
HS2
TO
HO
Ankle Angles [°]
DF(+): dorsiflexion
PF(-): plantarflexion

Figure S25

Figure S26

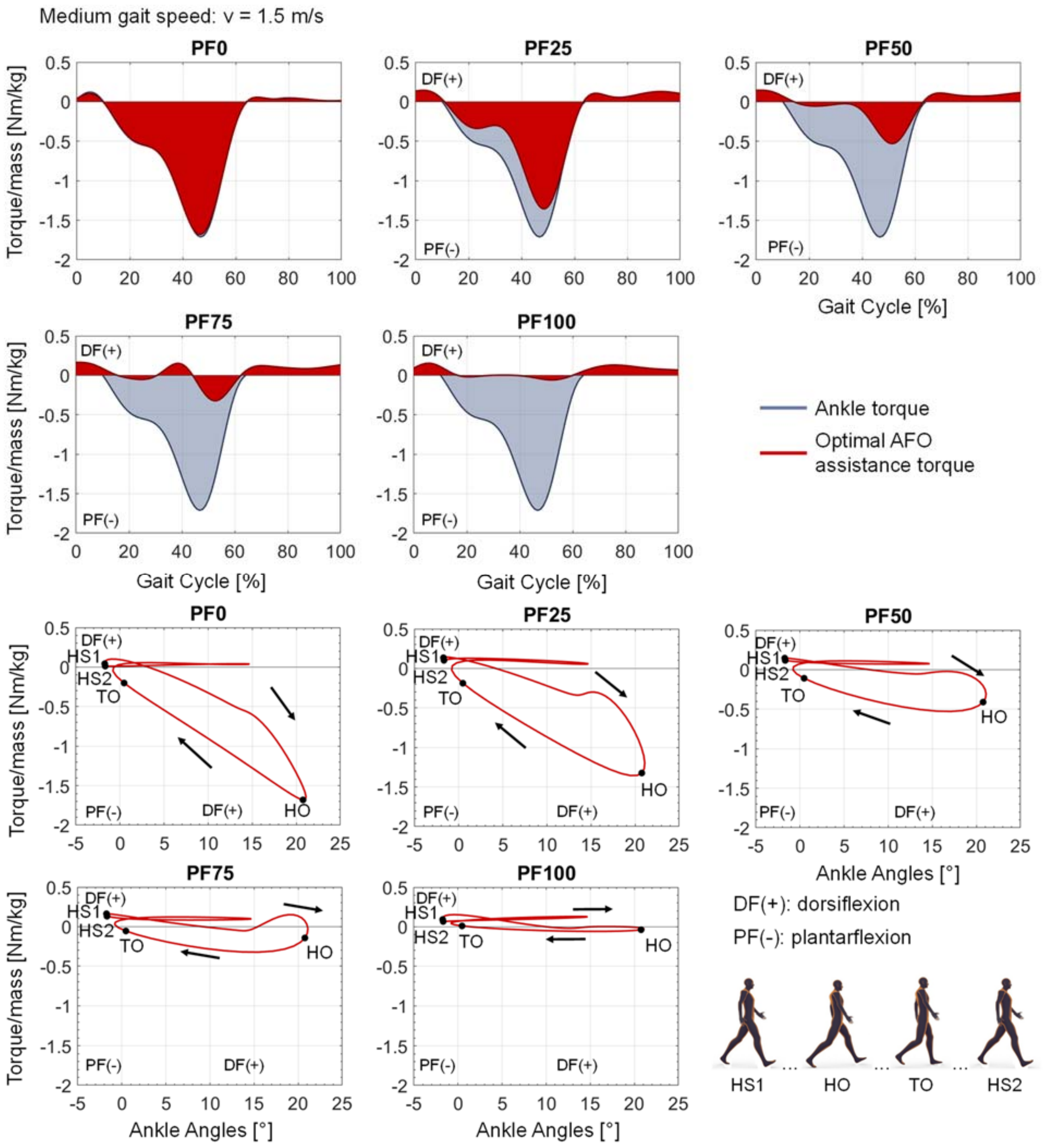

Medium gait speed: v = 1.5 m/s
PF0
PF25
PF50
PF75
PF100
Torque/mass [Nm/kg]
Gait Cycle [%]
DF(+)
PF(-)
Ankle torque
Optimal AFO assistance torque
HS1
HS2
TO
HO
Ankle Angles [°]
DF(+): dorsiflexion
PF(-): plantarflexion

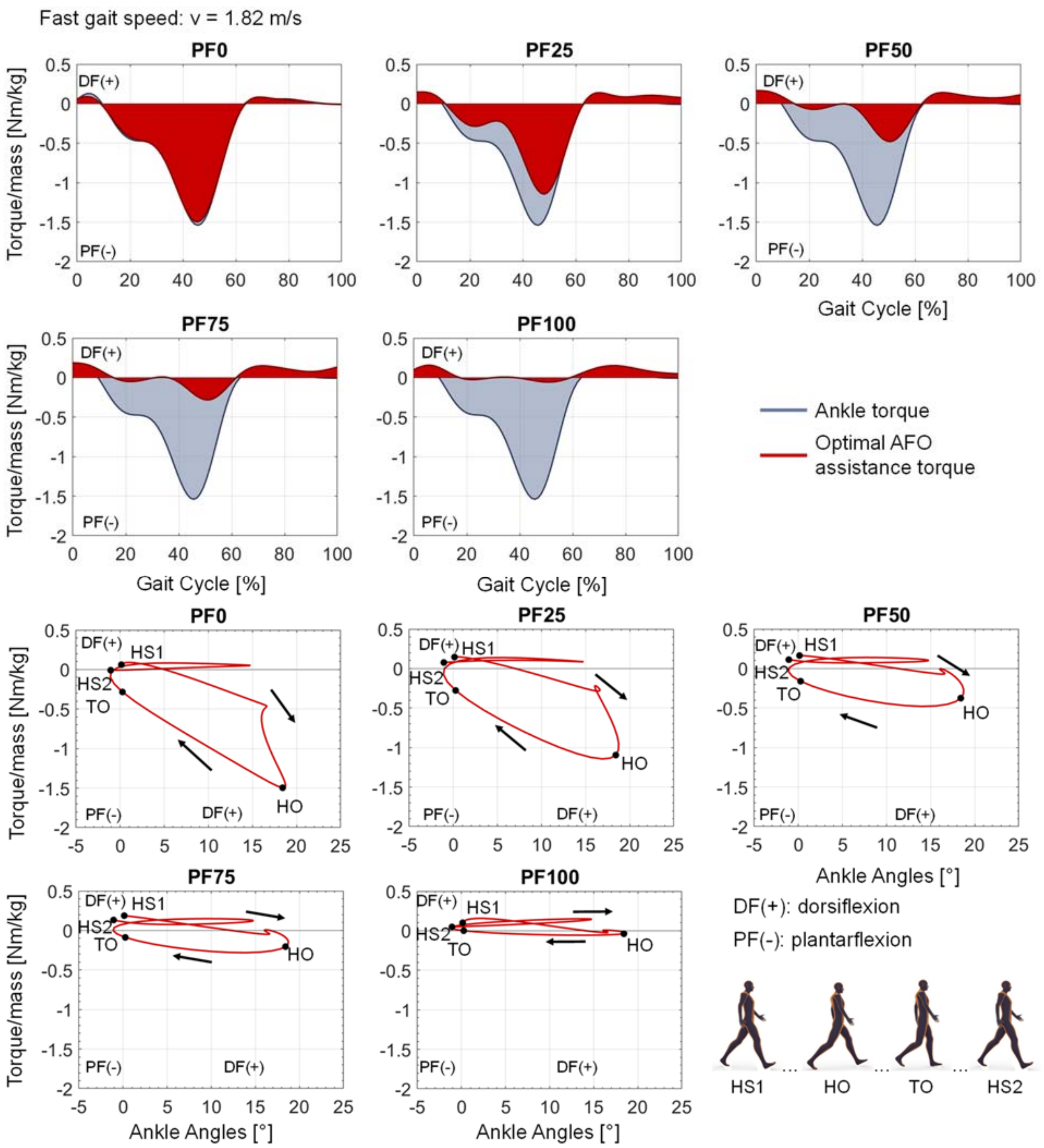

Fast gait speed: v = 1.82 m/s
PF0
PF25
PF50
PF75
PF100
Torque/mass [Nm/kg]
Gait Cycle [%]
DF(+)
PF(-)
Ankle torque
Optimal AFO assistance torque
HS1
HS2
TO
HO
Ankle Angles [°]
DF(+): dorsiflexion
PF(-): plantarflexion
HS1
HO
TO
HS2

Figure S27

Figure S28

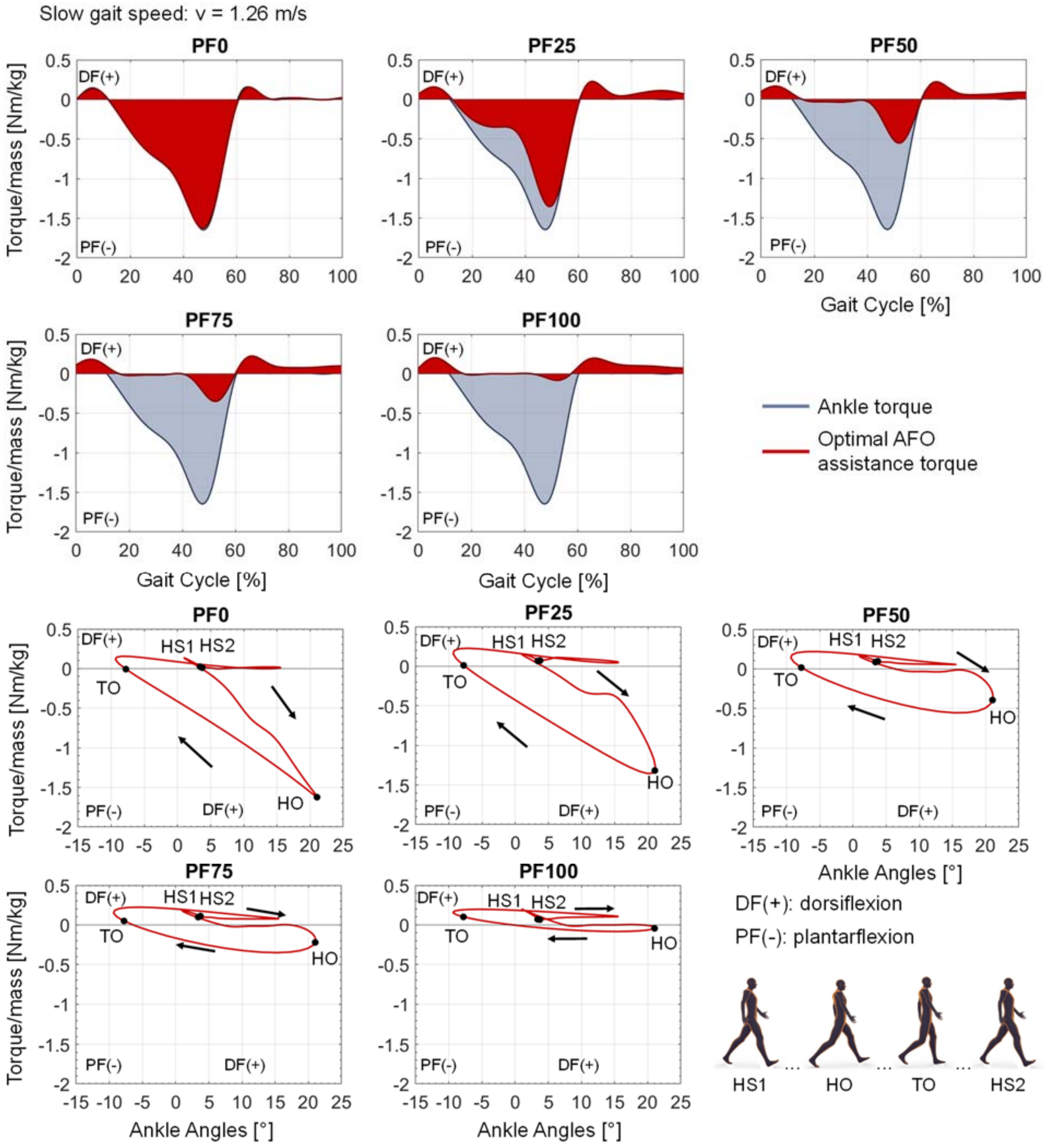

Slow gait speed: v = 1.26 m/s
PF0
PF25
PF50
PF75
PF100
Torque/mass [Nm/kg]
Gait Cycle [%]
DF(+)
PF(-)
Ankle torque
Optimal AFO assistance torque
HS1
HS2
TO
HO
Ankle Angles [°]
DF(+): dorsiflexion
PF(-): plantarflexion

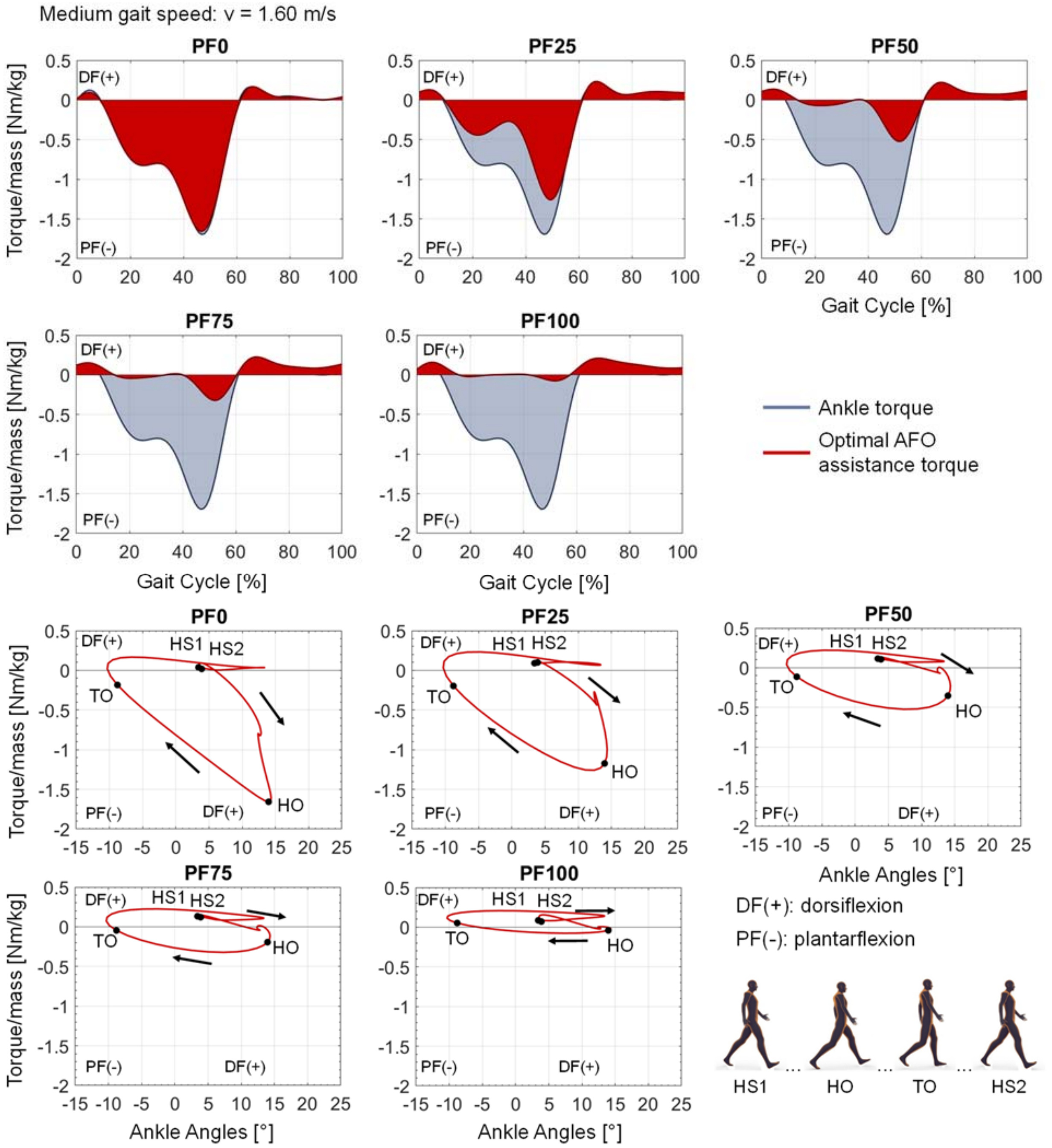
Medium gait speed: v = 1.60 m/s
PF0
PF25
PF50
PF75
PF100
Torque/mass [Nm/kg]
Gait Cycle [%]
DF(+)
PF(-)
Ankle torque
Optimal AFO assistance torque
HS1
HS2
TO
HO
Ankle Angles [°]
DF(+): dorsiflexion
PF(-): plantarflexion

Figure S29

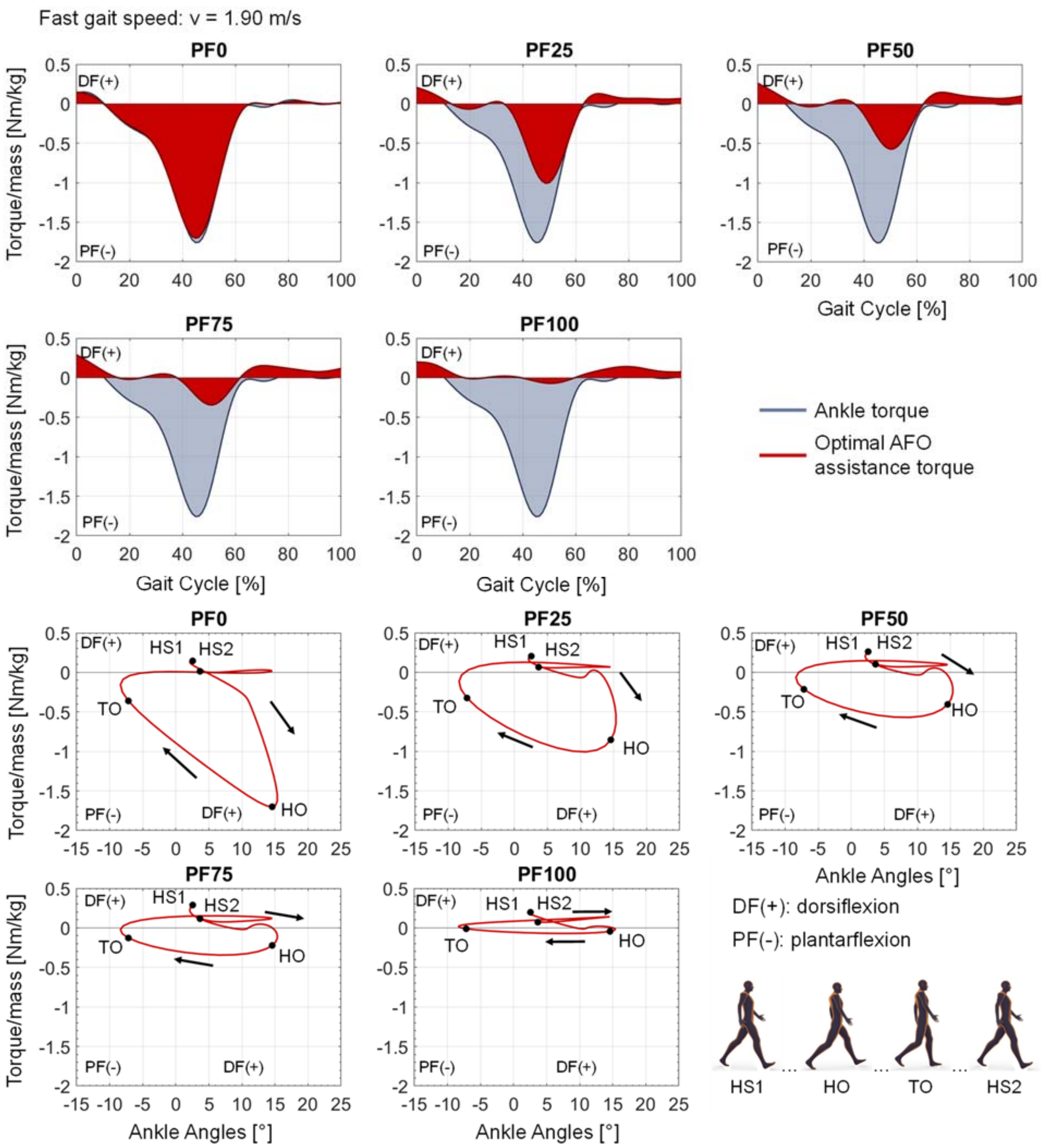
Fast gait speed: v = 1.90 m/s
PF0
PF25
PF50
PF75
PF100
Torque/mass [Nm/kg]
Gait Cycle [%]
DF(+)
PF(-)
Ankle torque
Optimal AFO assistance torque
Ankle Angles [°]
HS1
HS2
TO
HO
DF(+): dorsiflexion
PF(-): plantarflexion

Figure S30

Figure S31

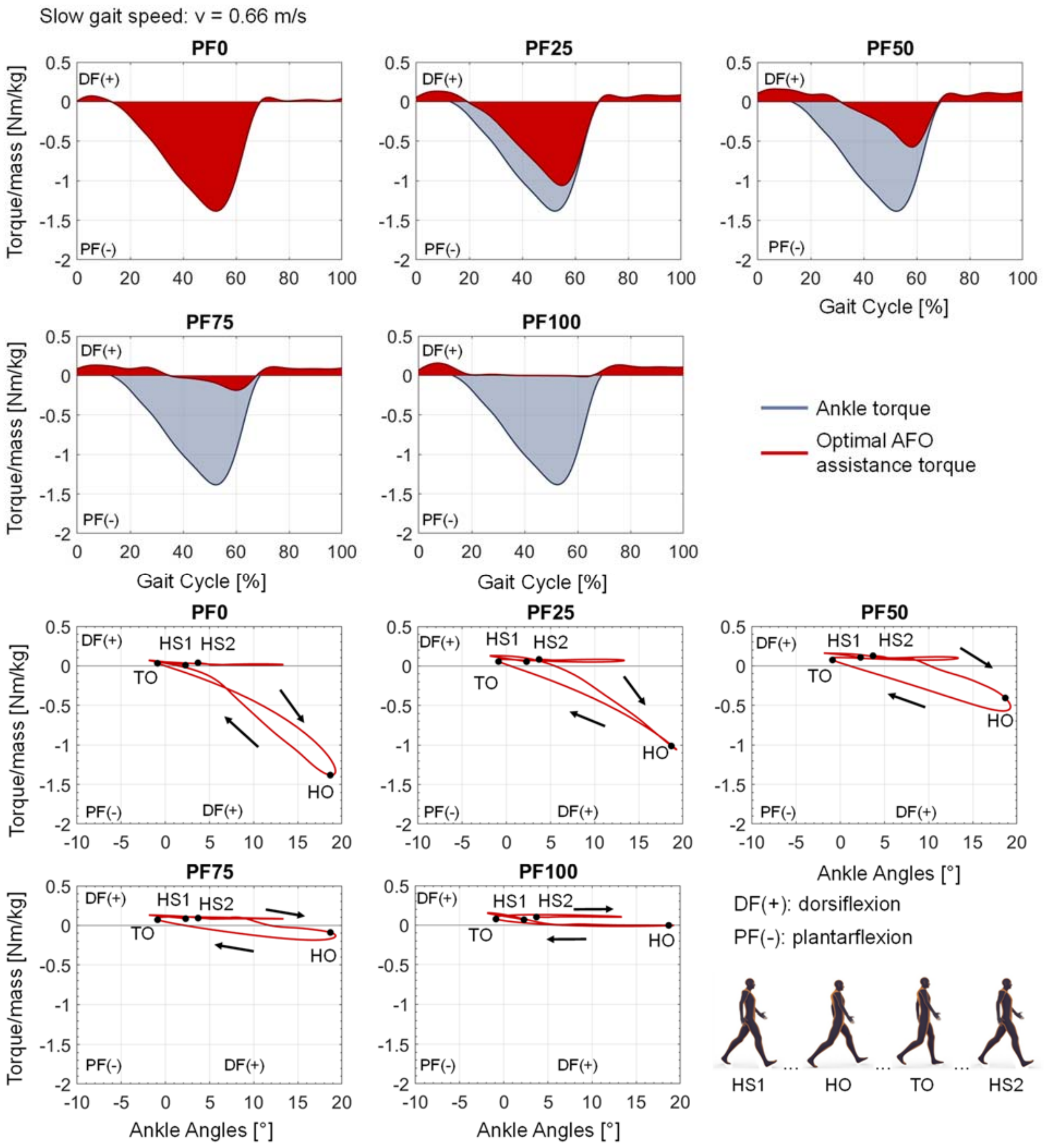
Slow gait speed: v = 0.66 m/s
PF0
PF25
PF50
PF75
PF100
Torque/mass [Nm/kg]
Gait Cycle [%]
DF(+)
PF(-)
Ankle torque
Optimal AFO assistance torque
HS1
HS2
TO
HO
Ankle Angles [°]
DF(+): dorsiflexion
PF(-): plantarflexion
HS1 ... HO ... TO ... HS2

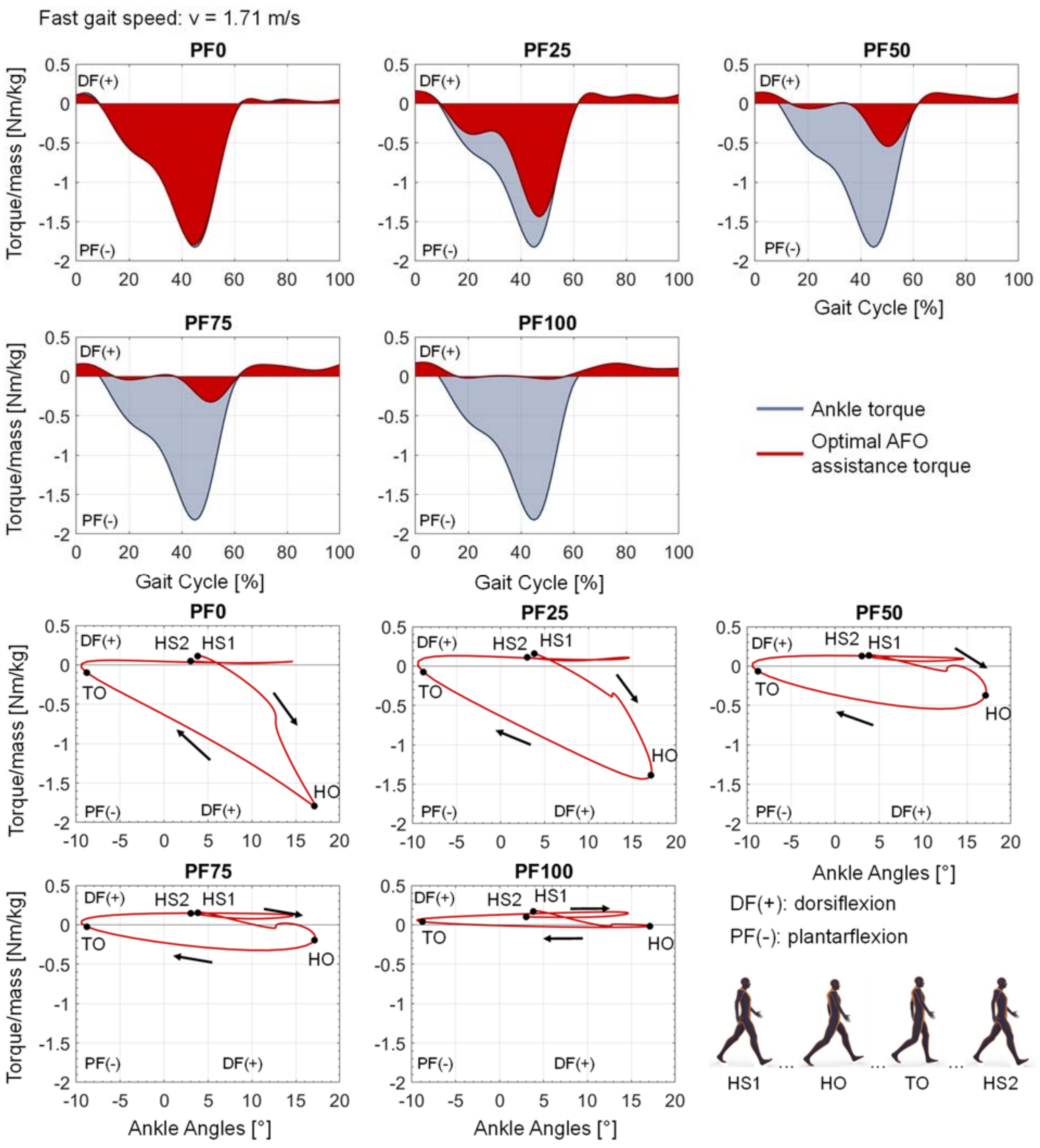

Fast gait speed: v = 1.71 m/s
PF0
PF25
PF50
PF75
PF100
Torque/mass [Nm/kg]
DF(+)
PF(-)
Gait Cycle [%]
Ankle torque
Optimal AFO assistance torque
HS2
HS1
TO
HO
Ankle Angles [°]
DF(+): dorsiflexion
PF(-): plantarflexion
HS1
HO
TO
HS2

Figure S32

Figure S33

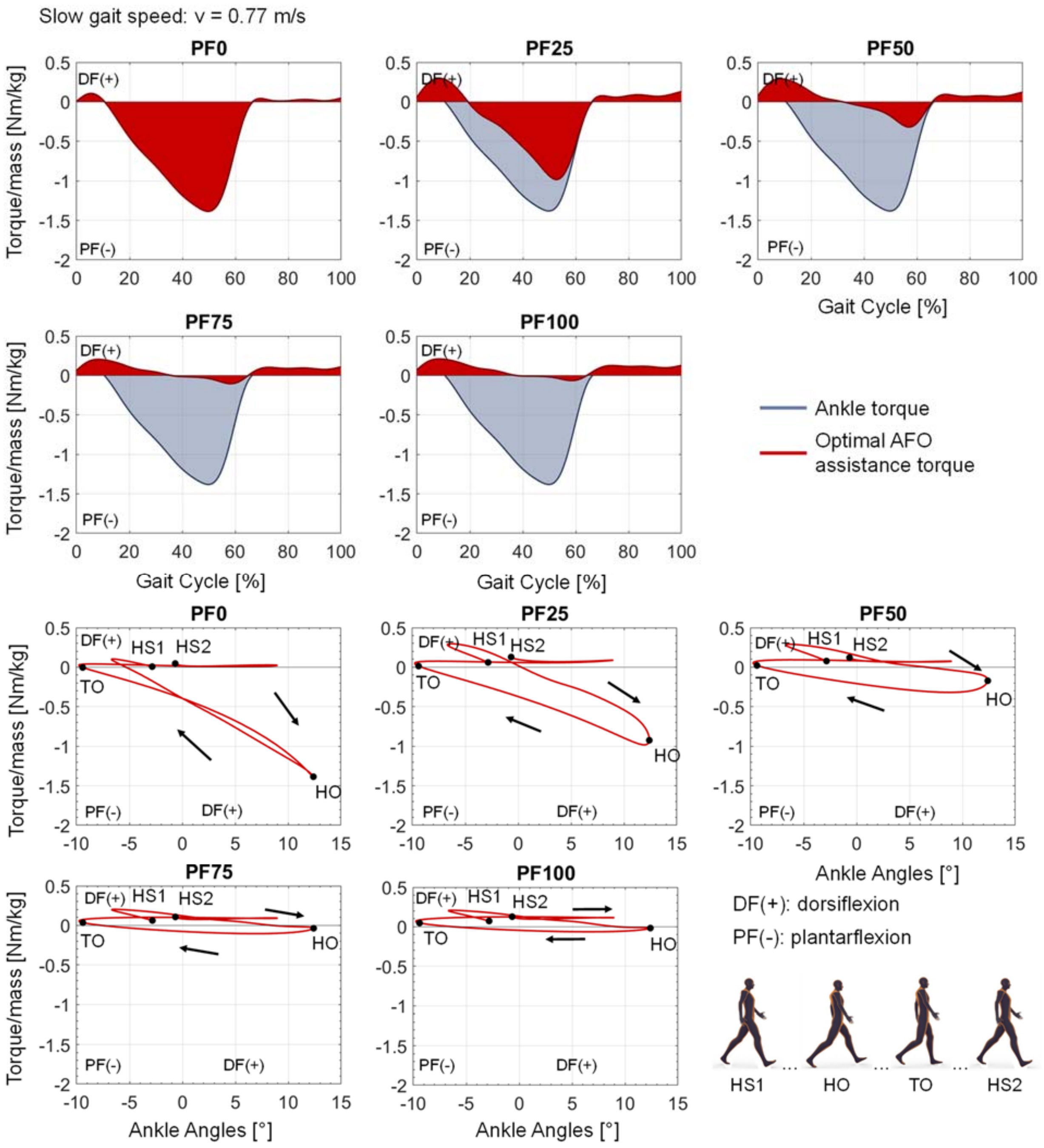

Slow gait speed: v = 0.77 m/s
PF0
PF25
PF50
PF75
PF100
Torque/mass [Nm/kg]
Gait Cycle [%]
DF(+)
PF(-)
Ankle torque
Optimal AFO assistance torque
HS1
HS2
TO
HO
Ankle Angles [°]
DF(+): dorsiflexion
PF(-): plantarflexion
HS1 ... HO ... TO ... HS2

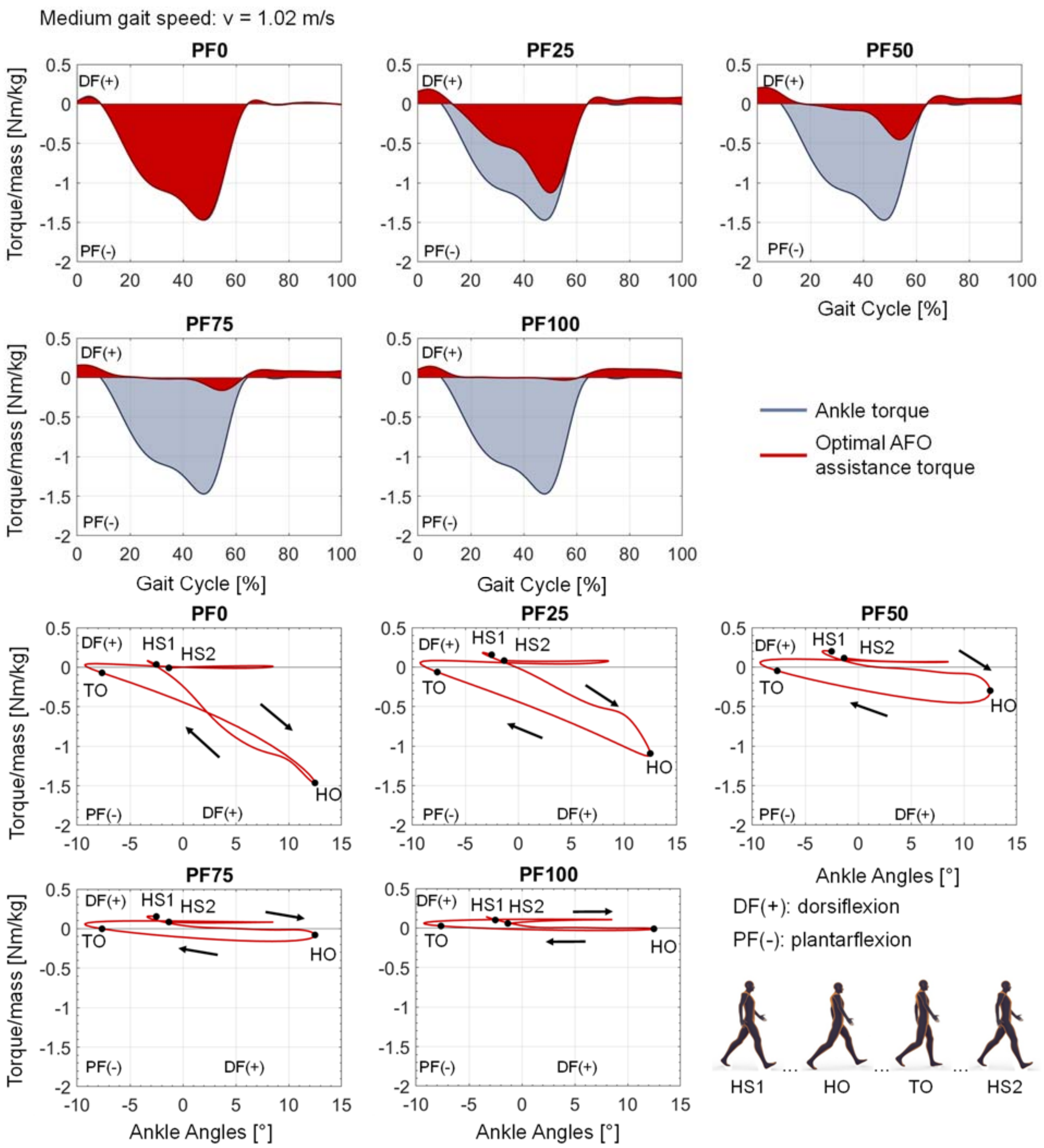
Medium gait speed: v = 1.02 m/s
PF0
PF25
PF50
PF75
PF100
Torque/mass [Nm/kg]
Gait Cycle [%]
DF(+)
PF(-)
Ankle torque
Optimal AFO assistance torque
HS1
HS2
TO
HO
Ankle Angles [°]
DF(+): dorsiflexion
PF(-): plantarflexion
HS1
HO
TO
HS2

Figure S34

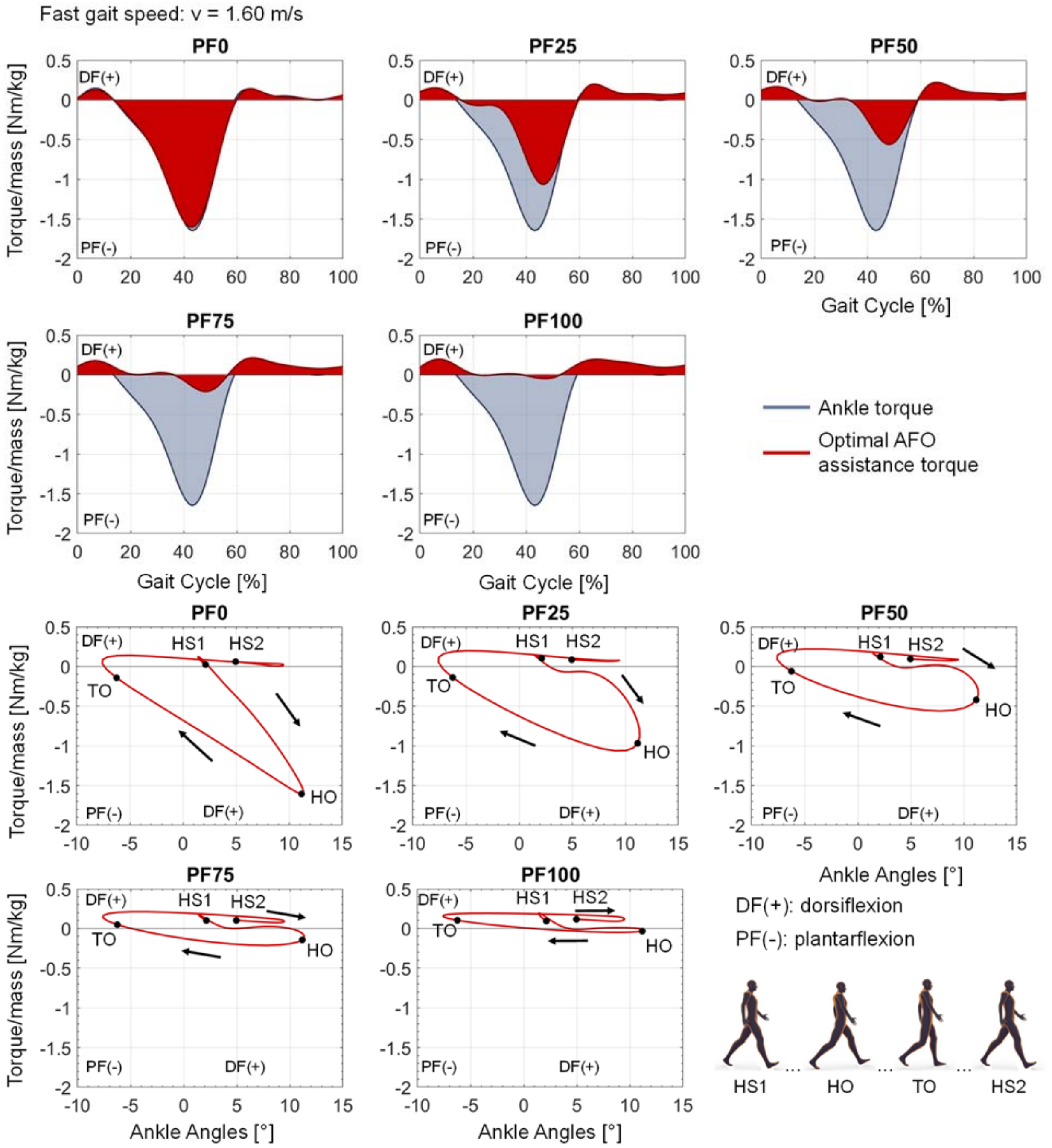

Fast gait speed: v = 1.60 m/s
PF0
PF25
PF50
PF75
PF100
Torque/mass [Nm/kg]
DF(+)
PF(-)
Gait Cycle [%]
Ankle torque
Optimal AFO assistance torque
HS1
HS2
TO
HO
Ankle Angles [°]
DF(+): dorsiflexion
PF(-): plantarflexion

Figure S35
